# Machine Learning Prediction and Interpretability Analysis of the Unconfined Compressive Strength of Alkali-Activated Stabilized Soft Soil Under Coupled Wetting–Drying Cycles and Acid Attack

**DOI:** 10.3390/ma19214472

**Published:** 2026-10-23

**Authors:** Zijian Huang, Yutong Pang, Junyi Zhang, Guo Hu, Jun Wu

**Affiliations:** 1 Mine Geology and Environment Academician and Expert Workstation, Ordos Institute of Technology, Ordos 017000, China; m405126263@sues.edu.cn; 2 School of Urban Rail Transportation, Shanghai University of Engineering Science, Shanghai 201620, China; m405124223@sues.edu.cn (J.Z.); huguo@sues.edu.cn (G.H.); 3 School of Civil Engineering, Shanghai Normal University, Shanghai 201418, China; cvewujun@shnu.edu.cn

**Keywords:** alkali-activated materials, stabilized soft soil, unconfined compressive strength, coupled wetting–drying and acid attack, machine learning, physics-informed neural network, SHAP, partial dependence

## Abstract

Predicting the unconfined compressive strength (UCS) of alkali-activated stabilized soft soil under coupled acid attack and wetting–drying cycles requires evaluation across correlated laboratory observations. This study analyzed 689 records from 53 complete mixtures, with seven material and environmental inputs, using four machine-learning models (DT, RF, SVM, and XGBoost) and four neural models (PINN, BPNN, CNN, and LSTM). Generalization to previously unseen mixtures was evaluated by five-fold outer GroupKFold with three-fold mixture-grouped tuning. Each original record contributed one out-of-fold prediction. The pooled grouped-CV $R^{2}$ values ranged from 0.5043 to 0.7605, with RMSEs of 0.3579–0.5148 MPa. XGBoost attained the highest point estimate ($R^{2}=0.7605$; RMSE = 0.3579 MPa), followed by SVM ($R^{2}=0.7362$; RMSE = 0.3756 MPa). PINN and its architecture-matched BPNN produced identical grouped-CV predictions and no demonstrated incremental benefit. A prospective penalty-activation diagnostic confirmed that soft penalties can affect training trajectories but did not reconstruct the archived model-selection runs. A separately tuned, five-step, structure-matched ablation found no additional predictive gain from preceding design covariates in CNN and LSTM. Historical fixed-configuration random-CV comparisons were removed because the original hyperparameter-selection provenance could not be verified. SHAP and partial dependence analyses describe model-learned associations, and SEM offers qualitative morphological context. These models provide preliminary screening aids within the tested materials and factor levels, not engineering design tools; independent batch and field validation remains necessary.

## 1. Introduction

### 1.1. Background and Significance

Transportation infrastructure in China continues to expand into coastal and inland soft-soil regions, where the quality of ground improvement directly affects the long-term serviceability and safety of subgrades, bridge abutments, and underground structures. Soft soils typically have high water contents, high compressibility, and low shear strength and are commonly stabilized using cementitious binders. In coastal environments, acid ingress can act jointly with wetting–drying cycles caused by groundwater-level fluctuations, exposing stabilized soil to repeated physical damage and chemical dissolution. Previous studies have documented progressive changes in strength and microstructure under acid attack and wetting–drying cycles at different pH levels [1,2,3].

Although Portland cement stabilization is well established, cement production carries a substantial energy and carbon burden. Alkali-activated materials (AAMs), which use industrial by-products such as slag and fly ash as precursors, are an important route toward lower-carbon binders. Their reaction products, mechanical properties, and durability are jointly governed by precursor chemistry, the activator system, and curing conditions [4,5,6,7]. In one-part systems, solid aluminosilicate precursors and a solid alkaline activator are premixed, requiring only the addition of water on site. This approach reduces the handling risks associated with transporting, storing, and preparing concentrated alkaline solutions and offers practical advantages for field applications [8].

### 1.2. Previous Research

The terminology used in this study reflects the chemistry of the binder. The experimental system combines ground granulated blast-furnace slag, with approximately 53.0% CaO, and low-calcium fly ash and is, therefore, a high-calcium slag–fly ash alkali-activated system. Reviews have shown that the reaction products and structure of alkali-activated binders depend on precursor calcium content and activation conditions. High-calcium slag systems commonly form binding phases dominated by C-(A)-S-H-type gels, whereas low-calcium fly ash systems favor aluminosilicate network structures [5,6,7]. Accordingly, the term “alkali-activated stabilized soft soil” is used consistently throughout this paper, and the mechanistic discussion is framed in terms of the products characteristic of AAM systems.

#### 1.2.1. Mechanical Properties of Alkali-Activated Stabilized Soil

Previous studies of slag–fly ash alkali-activated stabilized soft soil have shown that unconfined compressive strength (UCS) is sensitive to the slag-to-fly-ash ratio, activator dosage or activator-to-precursor ratio, and water-to-binder ratio. Zheng and Wu demonstrated that these three mixture variables substantially affect the early UCS of soft clay stabilized with a one-part GGBFS/FA binder. Their SEM, X-ray diffraction (XRD), and energy-dispersive X-ray spectroscopy (EDS) observations indicated that reaction products fill voids and bond soil particles [9]. Luo et al. further found that stabilizer dosage, the slag–fly ash proportion, and alkaline activator content jointly determine UCS and microstructural compactness in riverside soft soil [10]. Wu et al. examined activator parameters, slag content, and wetting–drying durability, confirming the influence of FA/slag alkali-activated systems on soft-soil strength and water stability [3]. A related review also highlighted the potential of blended GGBFS–FA alkali-activated binders as lower-carbon soil stabilizers, while emphasizing their strong dependence on material sources and mixture combinations [11].

#### 1.2.2. Durability Under Coupled Wetting–Drying Cycles and Acid Attack

Durability studies indicate that wetting–drying cycles and acidic media can both alter the strength and structure of alkali-activated stabilized materials, but their effects are not simply additive. Fakhrabadi et al. documented changes in the mechanical properties and microstructure of geopolymer-stabilized soil during wetting–drying cycles [12]. In cyclic tests at different pH levels, Ngo et al. found that strength generally decreased with increasing cycle number, although short-term strengthening could occur during early cycles under some pH conditions, indicating stage-dependent deterioration [2]. Regarding acid attack, the seminal work of Bakharev showed that geopolymer materials can exhibit favorable acid durability while still undergoing appreciable strength loss depending on the activation system [13]. Aiken et al. further reported that increasing slag content can reduce porosity while altering the stability of reaction products under sulfuric acid attack [14]. For one-part alkali-activated soft soil, Li et al. systematically examined the effects of GGBFS/FA ratio, acid type, pH, and immersion duration on UCS and microstructure [1]. Overall, quantitative evidence on simultaneous acid attack and wetting–drying exposure, particularly their multifactor interactions in stabilized soft soil, remains limited. This gap provides the durability context for the present modeling study.

#### 1.2.3. Data-Driven Prediction, Physical Knowledge Integration, and Interpretability

Machine learning and deep learning have been widely applied to strength prediction for cementitious materials, concrete, and stabilized soil. Hu et al. used RF, support vector regression (SVR), multilayer perceptron (MLP), gradient-boosting decision trees (GBDT), and XGBoost to predict UCS and optimize mixtures of slag–fly ash geopolymer-stabilized soil, demonstrating the potential of combining structured material variables with ensemble learning [15]. Recent explainable-learning applications have used tree ensembles and SHAP to interpret concrete strength [16,17], while a separate stabilized-soil UCS study combined machine learning with SHAP, partial dependence, and accumulated local effects [18]. Related deep-learning work on PET-fiber-reinforced one-part geopolymer-stabilized soil addressed ductility and feature interpretation [19]. These studies motivate a cautious examination of predictive and interpretive methods for this material system, without implying that their datasets or experimental protocols are identical. Classical PINNs incorporate physical equations into training constraints [20], whereas the broader field of physics-informed machine learning allows partial physical laws, conservation relationships, or structural priors to be integrated into learning [21]. Because no governing partial differential equation is directly available for the UCS prediction task considered here, this study formulates soft constraints using directional priors and actual experimental design levels rather than classical PDE residuals. For interpretation, SHAP provides a unified additive framework for attributing complex model predictions to input features [22].

### 1.3. Research Gaps and Questions

The literature reveals three related gaps that motivate the research questions addressed in this study.

The first gap concerns coupled environmental exposure. Although the mechanical properties of alkali-activated stabilized soils have been extensively investigated, most studies focus on a single environmental action. Deterioration under coupled acid attack and wetting–drying cycles remains insufficiently characterized, despite the relevance of these conditions to coastal soft-soil environments in eastern China.

The second gap concerns evaluation methodology. Studies of stabilized-soil strength prediction differ in their data-splitting protocols and approaches to model comparison. Random splitting is appropriate for assessing interpolation within the sampled design space. However, when one mixture is tested under multiple environmental conditions, the resulting samples are correlated, and structured cross-validation by mixture or group provides a more stringent assessment of generalization to unseen material combinations. Methodological studies have shown that random cross-validation can underestimate prediction error when spatial, temporal, or hierarchical dependencies are ignored [23]. The number of folds and the splitting strategy can also affect model selection [24]. Failing to distinguish these evaluation settings can, therefore, conflate accuracy within the sampled design space with generalization to unseen mixtures.

The third gap concerns the reliability of interpretation. Although SHAP and PDPs are widely used in material-property prediction, interpretations from a single model cannot establish that the inferred relationships are stable. They may instead reflect preferences imposed by that model’s structure.

Three research questions are, therefore, posed. RQ1: How do the eight machine-learning and deep-learning models differ in UCS prediction when evaluated on the same 689 records using a common primary protocol? RQ2: Do directional soft penalties demonstrate an additional physical-consistency or predictive benefit over an architecture-matched BPNN under the tested validation protocol? RQ3: Do different models identify consistent dominant material–environment relationships in terms of feature importance, effect direction, and response range?

### 1.4. Scope of the Study

To address these questions, this study investigates slag–fly ash alkali-activated stabilized soft soil through four tasks.

(1) A strength database containing 689 experimental records under coupled exposure is established and subjected to a data-quality audit.

(2) Four machine-learning models (DT, RF, SVM, and XGBoost) and four deep-learning models (PINN, BPNN, CNN, and LSTM) are developed within a unified evaluation framework. PINN incorporates known deterioration tendencies into its loss function through physical soft constraints.

(3) Predicted-versus-measured scatter plots, actual error-evolution plots, and R^2^ and RMSE metrics are reported for each model. SHAP, one-factor PDPs, and selected three-dimensional two-factor PDP response surfaces are used to assess consistency in dominant factors, effect directions, and combined effects.

(4) Activator-dependent response domains and representative SEM images at different Slag/FA ratios, pH levels, and cycling stages are used to discuss qualitative consistency between model responses and material deterioration, informing the joint consideration of mixture design and service environment.

## 2. Materials, Experimental Program, and Database

### 2.1. Raw Materials

**Figure 1:**
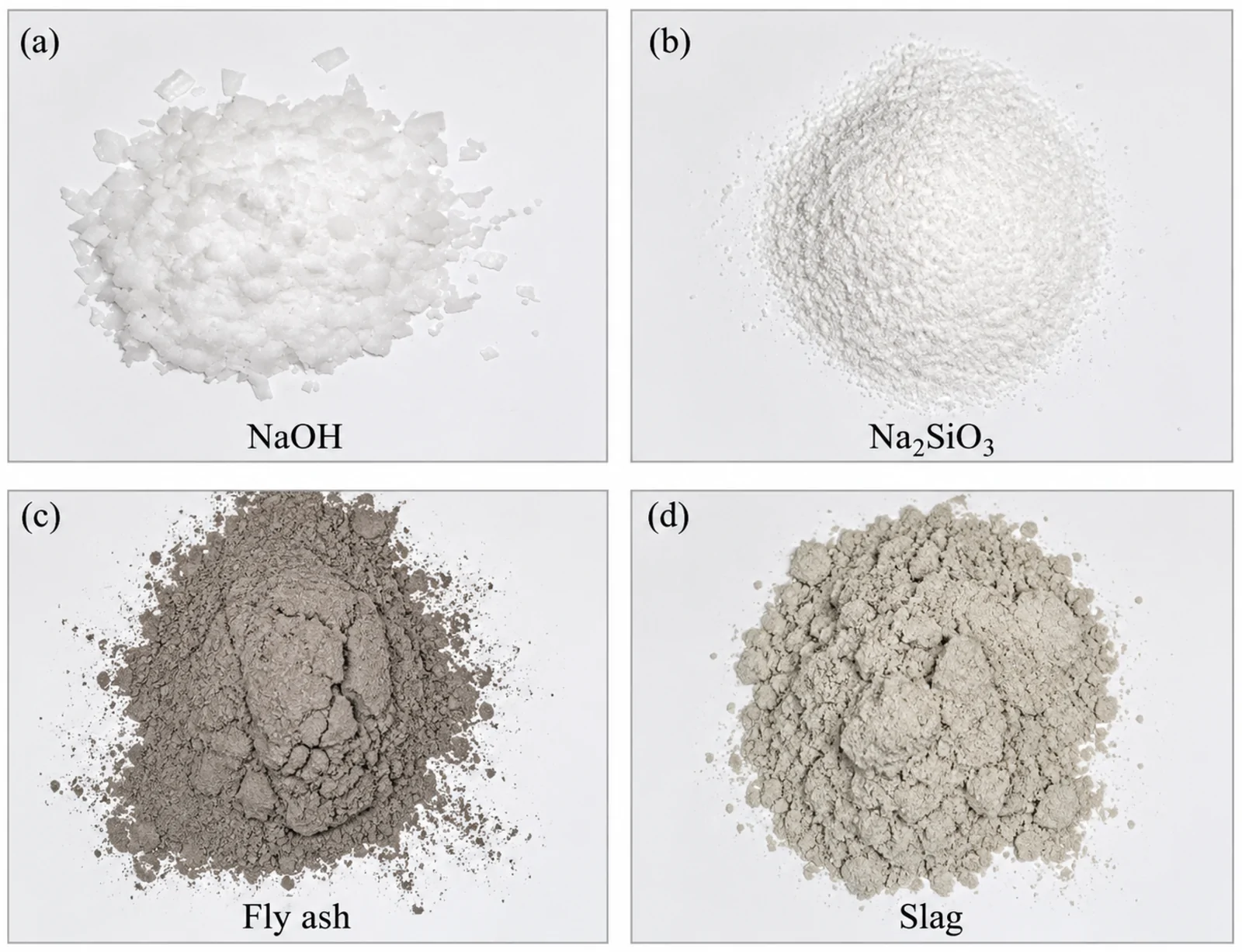
Raw materials: (**a**) NaOH; (**b**) solid sodium silicate; (**c**) fly ash and (**d**) slag.

The ground granulated blast-furnace slag (GGBFS) and low-calcium fly ash (FA) were obtained from a steel plant in Suqian, Jiangsu, and a power plant in Jiangyin, Wuxi, Jiangsu, respectively. The slag consisted primarily of CaO (approximately 53.0%) and SiO_2_ (approximately 37.3%). The fly ash was dominated by SiO_2_ (approximately 56.4%) and Al_2_O_3_ (approximately 29.4%), with only approximately 2.8% CaO, consistent with its classification as low-calcium fly ash. The soft soil was collected from a construction site in Putuo District, Shanghai. Its natural water content was approximately 50%, with a liquid limit of 43.3% and a plastic limit of 24.6%. Table 1 and Table 2 summarize the material properties. The slag, fly ash, and two solid alkaline activators are shown in Figure 1.

### 2.2. Alkaline Activators and One-Part Specimen Preparation

NaOH (reported purity at least 96%) and powdered sodium silicate are the two activators described in the source thesis [25]. The database code “Na_2_SiO_3_” denotes the silicate class, not a verified stoichiometry or silicate modulus. Although the thesis also uses “water glass”, its mixing description specifies a powder. Without a batch-specific SiO_2_/Na_2_O assay, neither the oxide composition nor the modulus of that powder can be confirmed. Figure 1 shows the raw material, and the corresponding sodium-silicate activator class is identified consistently in the SEM comparisons below.

A/P is a database-defined nominal activator-to-(slag + FA) ratio, not a batch-verified Na_2_O-equivalent dose. For NaOH (purity at least 96%), the stoichiometric Na_2_O-equivalent fraction per precursor mass is 0.775(A/P) times purity. This implies nominal ranges of 7.44–7.75%, 11.16–11.62%, and 14.88–15.50% for A/P = 0.10, 0.15, and 0.20, respectively; these are calculations, not measured batch dosages. The actual silicate-batch Na_2_O content and modulus are unverified, so equal A/P does not guarantee equal alkalinity or silica supply. Activator contrasts describe formulation classes, not iso-alkalinity. Specimens were molded in three layers, sealed, demolded after about 24 h, and cured for 28 d at 20 ± 2 °C and at least 95% RH.

**Figure 2:**
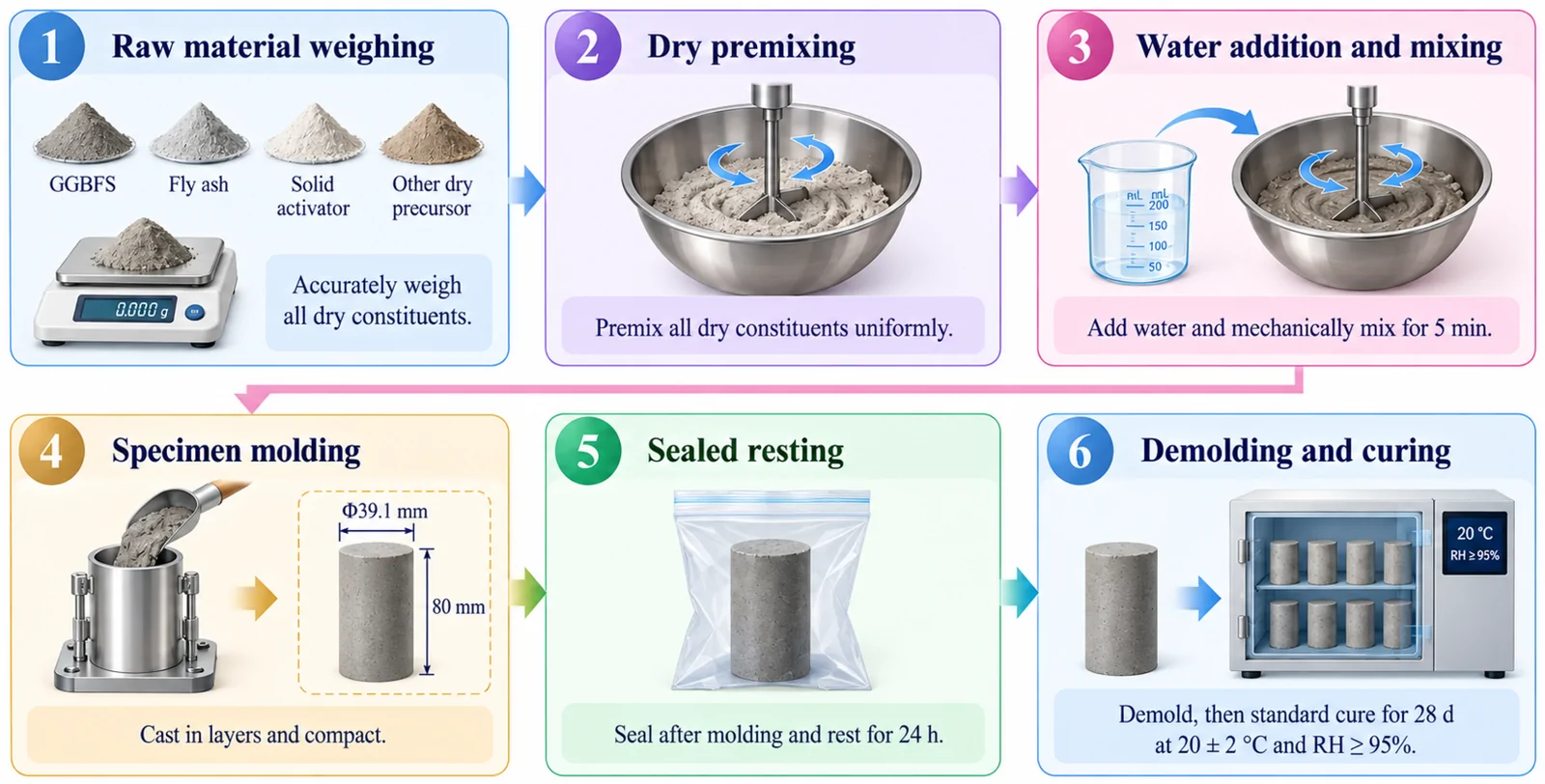
One-part specimen preparation procedure. Arrows indicate the order of the six depicted preparation operations and the direction of mixing or material transfer.

The six-step one-part preparation schematic is shown in Figure 2.

### 2.3. Mixture Design and Coupled Exposure Program

The design varied Slag/FA (2.34, 4, 9), activator (NaOH or sodium silicate), and database-defined A/P (0.10, 0.15, 0.20) and W/B (0.5, 0.7, 0.9); 53 of 54 mixes were observed (Table 3). A/P nominally refers to activator/(slag + FA). The source protocol prewetted dry soil with 10–15% water by soil mass. As binder mass relative to dry soil and whether prewetting water enters W/B are not recorded, neither an absolute binder dosage nor a verified physical W/B denominator can be reported.

The source protocol prescribed 7 d drying (23 ± 2 °C, 65 ± 5% RH) plus 7 d immersion in single-use sulfuric acid (pH 2, 4, or 6) per cycle; solution covered specimens by 20 mm and pH was checked daily (±0.05). Table 4 summarizes the recorded exposure stages and baseline.

### 2.4. Material Pretreatment, Strength Testing, and SEM Observations

**Figure 3:**
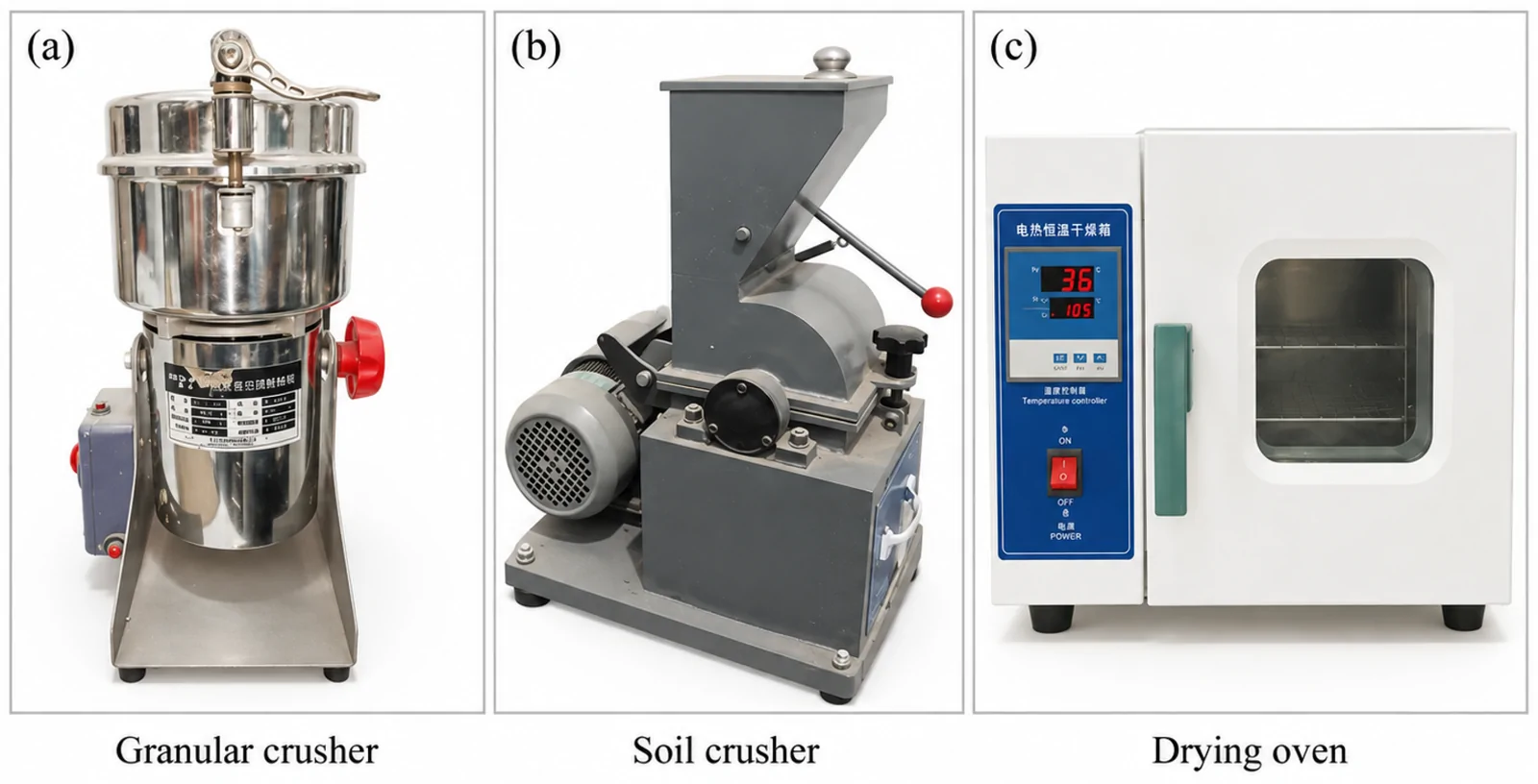
Material pretreatment equipment: (**a**) granular crusher; (**b**) soil crusher; (**c**) drying oven. Text printed in Chinese on the device nameplates is instrument labeling, not an experimental variable.

Soil was crushed, sieved (2 mm), and oven-dried (105 °C, 8 h; Figure 3). The source thesis specifies nominal 50-mm-diameter, 80-mm-high cylinders (Figure 2), cured for 28 d before cyclic exposure. The nominal UCS ages for N = 0, 1, 5, 10, 15 are 28, 42, 98, 168, 238 d after casting, assuming uninterrupted 14-d cycles; actual specimen-level dates are unverified. The related UCS protocol cites GB/T 50123-2019 [26], dimensional checks, and displacement-controlled loading at 1.0 mm/min; UCS equals peak force divided by measured area. Specimen-specific dimensions likewise require batch-log confirmation.

The related protocol specifies three parallel specimens per condition, averaged for UCS. The 689-row dataset retains means only, so within-condition SD, CV, and replicate error bars cannot be estimated. Model RMSE/MAE measure prediction error, not test scatter. The original test logs are needed to verify replicate counts and quantify laboratory variability.

**Figure 4:**
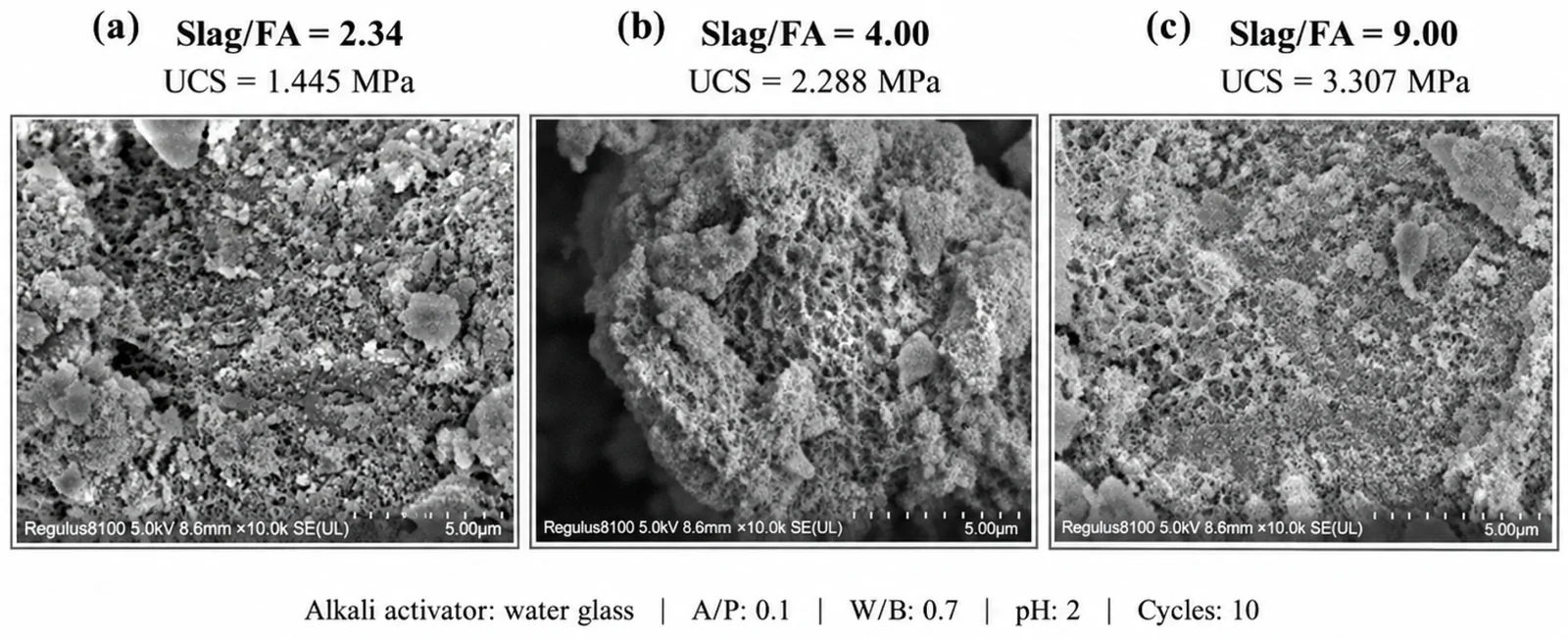
SEM comparison of representative specimens at different Slag/FA ratios for the solid sodium-silicate activator class.

(1) Variation in Slag/FA. Three sodium-silicate-activated specimens were selected with A/P = 0.10, W/B = 0.7, pH = 2, and Cycle = 10 held constant. Only Slag/FA varied, taking values of 2.34, 4, and 9; the corresponding UCS values were 1.445, 2.288, and 3.307 MPa, respectively (Figure 4).

This image series compares matrix continuity, particle bonding, and open-pore morphology as precursor composition changes. Section 4.9 examines whether these observations are directionally consistent with the Slag/FA PDP response.

**Figure 5:**
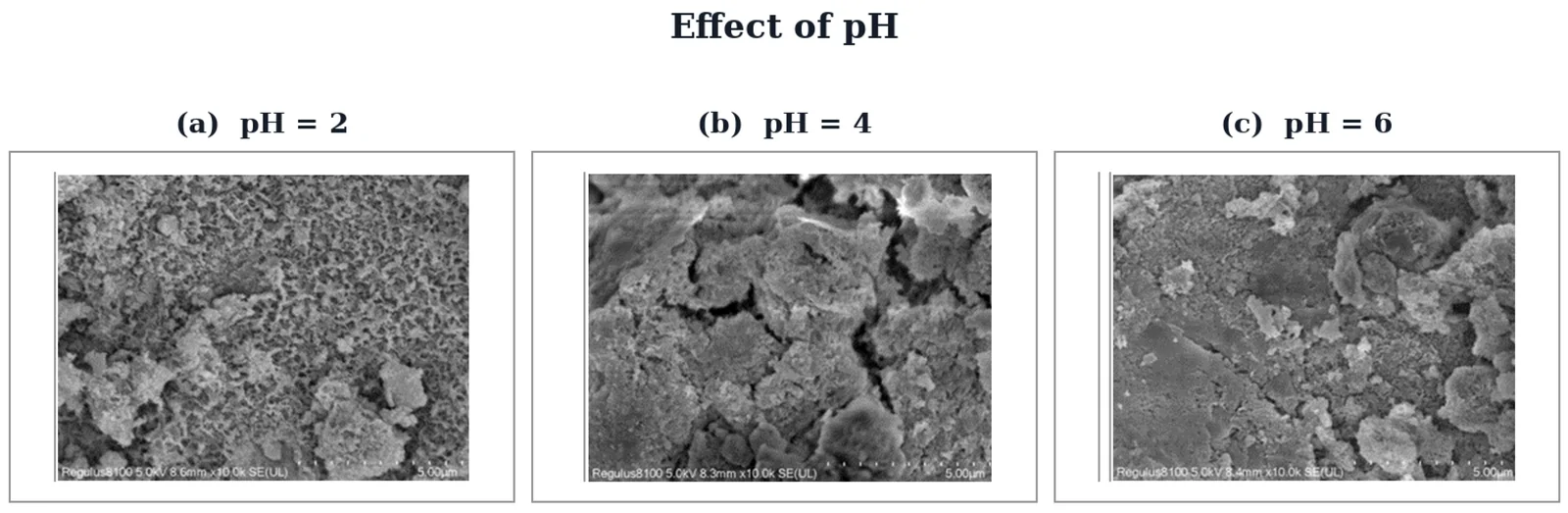
SEM comparison of representative specimens at different pH levels: (**a**) pH 2; (**b**) pH 4; (**c**) pH 6.

(2) Variation in pH. Representative specimens exposed at pH 2, 4, and 6 were selected to compare local pores, cracks, and matrix continuity under different acidity levels (Figure 5).

This image series illustrates relative morphological differences among acidic environments. Because SEM observations are sensitive to local field-of-view heterogeneity, they are not used to establish a strictly monotonic quantitative relationship.

**Figure 6:**
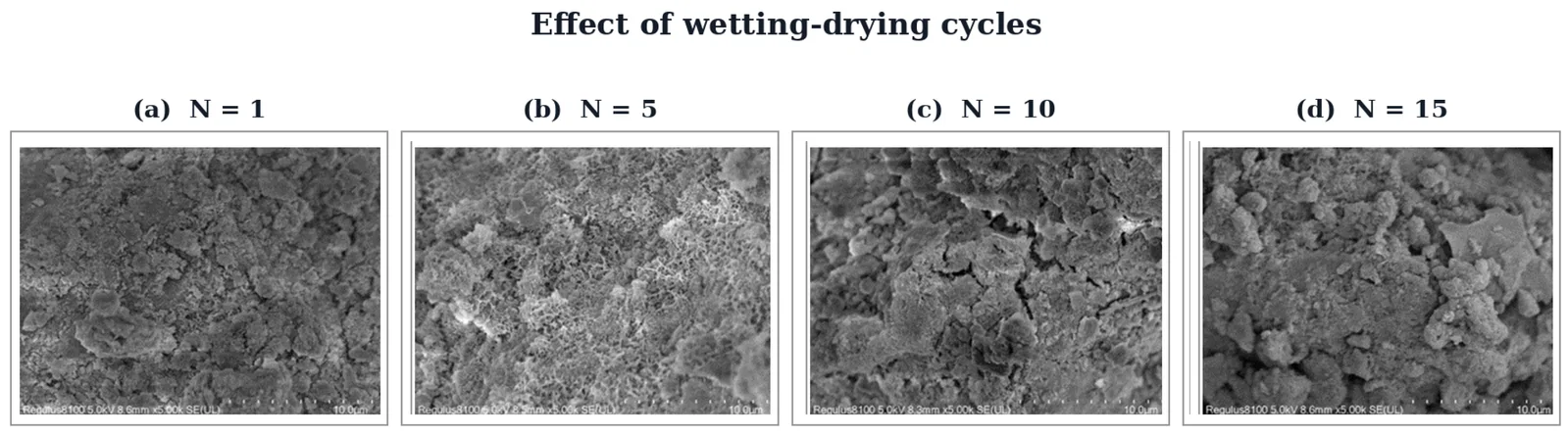
SEM comparison of representative specimens at different wetting–drying stages: (**a**) 1 cycle; (**b**) 5 cycles; (**c**) 10 cycles; (**d**) 15 cycles.

(3) Variation in wetting–drying cycle number. Representative specimens at N = 1, 5, 10, and 15 cycles were selected under the corresponding exposure conditions to compare pore development, cracking, and localized loosening or fragmentation as cycling accumulated (Figure 6).

Representative SEM views describe matrix continuity, voids, particle gaps, and cracks under selected conditions. Their limited fields do not quantify specimen-wide porosity or establish reproducible group differences. The images cannot identify C-(A)-S-H phases or prove gel formation, acid dissolution, or mechanisms of UCS loss. Section 4.9 evaluates qualitative agreement, alternatives, and limitations.

### 2.5. Database Composition and Quality Audit

The 689 UCS rows analyzed here were transcribed from the numbered laboratory-data appendix of Zhang’s 2026 MSc thesis [25], not generated as a new independent test campaign. The same 689 observation-level values are submitted as machine-readable Supplementary Data S1, with variable definitions and data-quality notes in File S1. Row-wise checks against that appendix found exact agreement in the mixture factors, activator, pH, cycle labels, and UCS values for all 689 entries. The thesis abstract describes 688 valid cases, but the appendix actually enumerates 689 rows; the present database follows the appendix with unresolved source-label issues disclosed. Its nominal 54-mix factorial grid contains 53 observed mixes; the missing combination is S/F = 2.34, Na_2_SiO_3_, A/P = 0.15, and W/B = 0.9, and no missing values were imputed.

Prior studies [1,9,19] used related one-part geopolymer-stabilized soil platforms, but examined acid immersion, early-age curing strength, and PET-fiber ductility, respectively, rather than the present complete wetting–drying/acid-exposure grid. Because cross-study specimen identifiers and original laboratory logs are unavailable, some underlying material or measurements may overlap; independence from those studies cannot be asserted. The thesis also analyzed machine-learning prediction and SHAP/PDP; this article extends, rather than originates, that work by applying nested mixture-grouped validation across eight learners and auditing physical constraints and sequence inputs.

The source data contain 53 zero-cycle baselines with physically undefined pH, encoded with a numerical placeholder and Exposure = 0. Two mix-pH paths have contradictory cycle labels: samples 385/386 (Mix 30, pH 4, N = 15; UCS 2.8158/2.0333 MPa) and 394/395 (Mix 31, pH 6, N = 10; UCS 3.2942/3.0042 MPa), each with one missing alternative cycle position. The original labels and both different UCS measurements are retained, flagged, and not interpreted as confirmed independent replicates; determining whether they are transcription errors requires the underlying specimen logs. Model validation uses all 689 rows, grouped by Mix_ID. For CNN and LSTM, valid-position masks handle incomplete sequences: missing positions do not contribute to the loss, while the remaining valid observations are retained. The primary evaluation consequently covers the same 689 observations for all models. Table 5 defines the modeling variables and derived audit field.

Table 6 lists the source-data consistency checks and their treatment in the analysis.

The audit categories are not mutually exclusive: two of the 53 baseline records (Sample_ID 118 and 157) also belong to the seven high-UCS records. Therefore, 623 of the 689 records have no audit flag; all 689 observations were retained for modeling.

Generative AI was used solely to assist with the graphical visualization of Figure 2. GPT-6 (OpenAI) was used to assist in preparing the schematic presentation, while the scientific content, technical relationships, labels, and interpretation shown in the figure were specified, reviewed, and revised by the authors. No generative AI tools were used to generate or modify experimental data, model outputs, statistical results, or other quantitative results. All other figures were generated using Python based on the experimental or computational data of this study.

## 3. Machine-Learning Methods

### 3.1. Overall Framework

**Figure 7:**
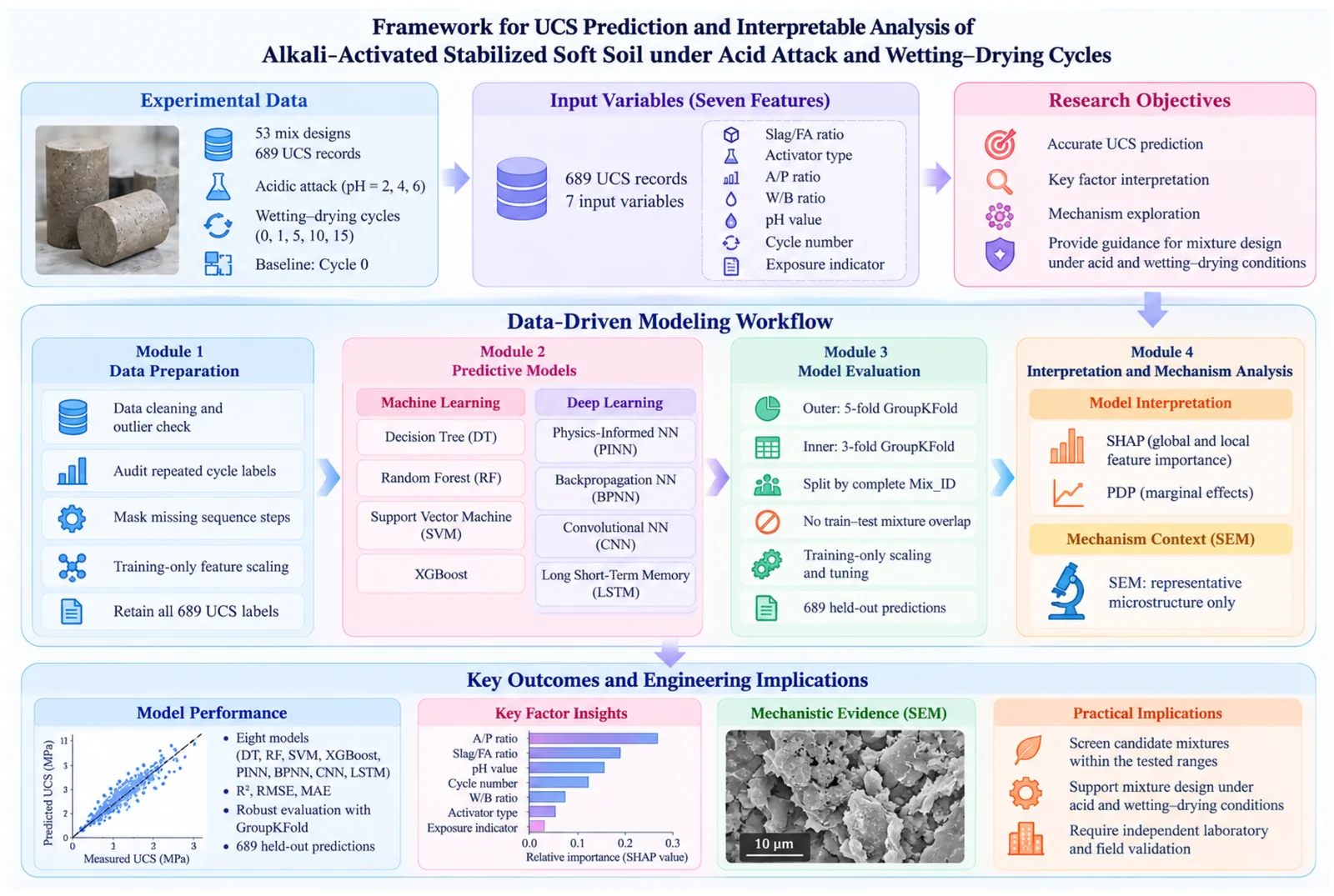
Overall framework for UCS prediction and model interpretation. Arrows indicate the sequential transfer from recorded data to model inputs and objectives, through the analysis workflow, to preliminary outcomes and validation needs.

The study comprises database construction and quality auditing, preprocessing and unified validation, training of eight models, predictive-performance comparison, supplementary robustness analysis, SHAP/PDP interpretation, and a discussion of mechanistic consistency between representative SEM observations and model interpretations (Figure 7). The main evaluation uses nested GroupKFold on 53 complete mixture groups; historical fixed-configuration random-CV results are excluded from the quantitative model comparison because their original tuning provenance could not be verified. An architectural control between PINN and BPNN isolates the incremental effect of physical soft constraints, and cross-model interpretation assesses the stability of dominant factors.

### 3.2. Preprocessing and Unified Validation Strategy

The main eight-model comparison uses seven inputs (S/F, activator, A/P, W/B, pH, Cycle, Exposure) with nominal A/P and W/B definitions. Exposure equals 1 if and only if Cycle >0 (689/689 observations): it supplies no independent information, and Cycle = 0 already identifies the baseline, whose pH = 7 is only a placeholder. A matched six-input XGBoost ablation without Exposure is reported in Section 4.3; Table 7 retains the shared seven-input results.

Generalization to unseen mixtures was assessed using a common nested mixture-grouped protocol. In the outer five-fold GroupKFold, every observation belonging to one Mix_ID was assigned to the same held-out fold. Within each outer training partition, three-fold GroupKFold selected hyperparameters using validation MAE; the outer test mixtures were never used for model selection, early stopping, or preprocessing. SVM used a scaler within its fitting pipeline; neural-network transformations were fitted exclusively on the corresponding training data. Tree-based methods used the original input units.

The deep-learning models used internal group-separated validation to select training duration; final test predictions averaged three prespecified random seeds (42, 52, and 62). CNN/LSTM represented 159 deterioration sequences and mapped valid outputs back to all 689 individual observations, masking missing positions and weighting the shared baseline only once. Performance was calculated from the pooled 689 outer-fold out-of-fold (OOF) predictions. Previously reported fixed-configuration random five-fold scores were omitted from the present quantitative comparison because independence of their hyperparameter selection could not be established.

**Figure 8:**
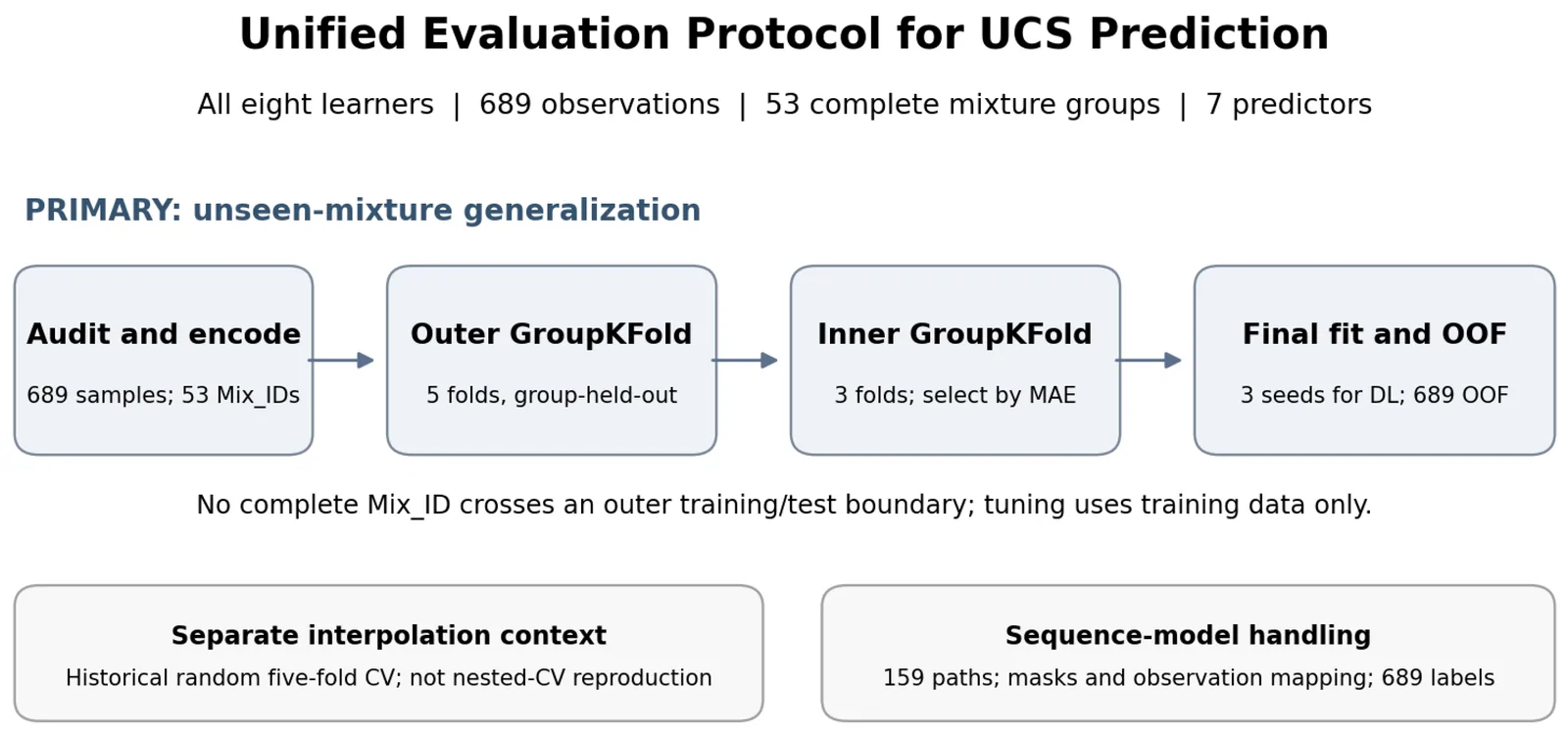
Unified nested GroupKFold assessment for all eight models; only mixture-grouped outer-test performance is used for the principal comparison.

The nested grouped-validation workflow is illustrated in Figure 8.

### 3.3. Machine-Learning Models

DT, RF, radial-basis-function SVR, and XGBoost [27,28,29,30,31] used identical outer five-fold GroupKFold splits by Mix_ID. Within each outer training set, an exhaustive grid search with inner three-fold GroupKFold minimized MAE. Scaling and parameter selection excluded the held-out outer mixtures; the selected estimator was then refit on that outer training set. Search grids and optimal configurations for outer folds F1–F5 are given below.

DT (depth, leaf): grid depth = {None,3,5,8}, leaf = {1,2,4}; selected (5,2), (5,1), (8,4), (5,4), (5,1).

RF (trees, depth, leaf): grid trees = {200,400}, depth = {None,6,10}, leaf = {1,2}; selected (400,6,1), (400,6,2), (400,None,2), (400,None,2), (200,None,2).

SVR (C, gamma, epsilon): grid C = {1,10,100}, gamma = {scale,0.1,0.5}, epsilon = {0.05,0.10}; selected (1,0.1,0.1), (1,0.1,0.1), (1,0.1,0.05), (1,0.1,0.05), (1,0.1,0.1).

XGBoost (trees, lr, depth, subsample): grid trees = {200,400}, lr = {0.05,0.10}, depth = {3,5}, subsample = {0.8,1.0}; selected (200,0.10,3,1.0), (200,0.05,3,0.8), (400,0.05,3,0.8), (400,0.10,3,0.8), (200,0.05,3,0.8).

### 3.4. Deep-Learning Models

The deep learners were BPNN, PINN, 1D-CNN, and LSTM, drawing on established backpropagation, convolutional, and LSTM architectures [32,33,34]. BPNN and PINN shared the 7-128-(3 × 128 residual blocks)-64-1 SiLU/AdamW backbone (residual connections, SiLU, and decoupled weight decay are described in [35,36,37]); only PINN included soft penalties (Section 3.5). CNN and LSTM used five-step sequences with masks, weighted shared baselines, and duplicate-record mapping (Section 3.6).

All networks used AdamW (weight decay $1\times 10^{-5}$), up to 250 epochs, and patience 40. Learning rates were 0.001 (BPNN/PINN) or {0.001, 0.003} (CNN/LSTM), selected by inner grouped MAE. Within each inner fit, a mixture-disjoint subset guided early stopping. The median of three inner selected epochs was used for outer refits; held-out predictions from seeds 42, 52, and 62 were averaged.

**Figure 9:**
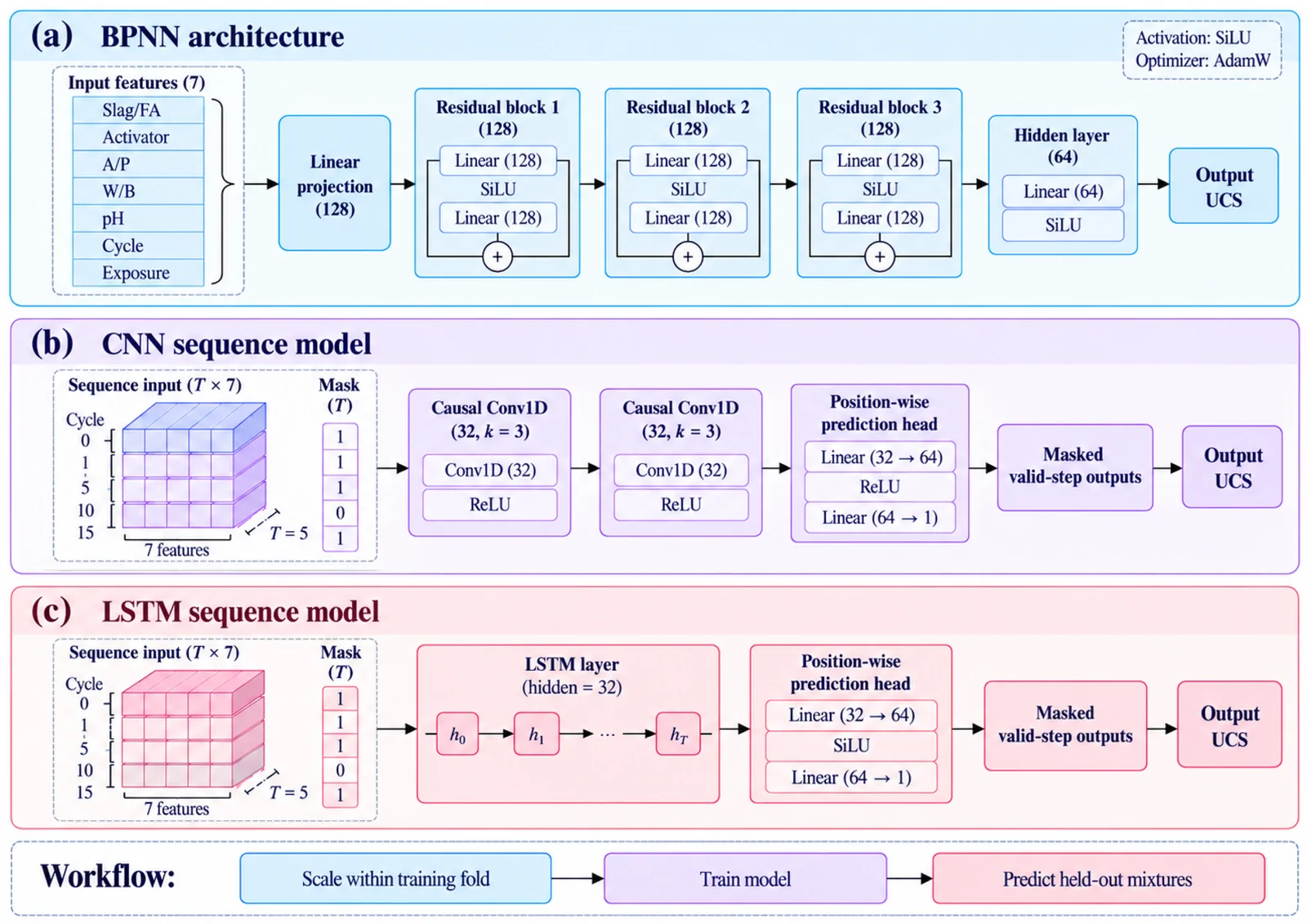
Schematic architectures of BPNN, 1D-CNN, and LSTM.

Software: Python 3.12.3, PyTorch 2.14.1+cpu, NumPy 2.5.2, pandas 3.0.6, scikit-learn 1.9.1, and XGBoost 3.4.1. Figure 9 illustrates the architectures.

Unlike the pointwise BPNN, the CNN and LSTM process ordered exposure-condition sequences constructed from independent specimens at different wetting–drying cycle levels.

### 3.5. Physics-Informed Neural Network and Physical Soft Constraints

PINN and BPNN predict UCS from the same seven inputs (S/F, activator, A/P, W/B, pH, Cycle, Exposure); UCS is never an input. They share the architecture and training protocol, while PINN alone adds directional soft penalties. These are finite-difference material priors rather than residuals of a governing PDE [20,21].

Let u(x) denote predicted UCS (MPa), $s_{y}$ the training-target standard deviation, and τ=0.02 MPa the tolerated strength increase. For neighboring levels a<b, define $D\left(a,b\right)={\mathrm{mean}}_{x}\left\{\max{\left[0,\left(u\left(x_{b}\right)-u\left(x_{a}\right)-\tau \right)/s_{y}\right]}^{2}\right\}$, where each paired anchor differs only in the chosen factor. Model inputs and anchors are taken from training mixtures and standardized using training-only statistics.

Shape-constrained ML can enforce globally monotone functions [38], whereas the present design-level squared-hinge formulation does not guarantee global monotonicity. The water/binder penalty is $L_{\mathrm{WB}}=\left[D_{\mathrm{WB}}\left(0.5,0.7\right)+D_{\mathrm{WB}}\left(0.7,0.9\right)\right]/2$ at the recorded W/B levels. The cycle penalty is $L_{\mathrm{Cycle}}=\left[D_{\mathrm{Cycle}}\left(1,5\right)+D_{\mathrm{Cycle}}\left(5,10\right)+D_{\mathrm{Cycle}}\left(10,15\right)\right]/3$; the 0→1 transition is omitted to allow early gains [2]. The nonnegativity penalty is $L_{\mathrm{pos}}={\mathrm{mean}}_{x}\left\{\max{\left[0,-u\left(x\right)/s_{y}\right]}^{2}\right\}$, evaluated on distinct acid-exposed training inputs. These use squared target-scaled excess rather than derivative penalties.

For efficiency, up to 120 distinct training anchors per directional context were sampled using seed 123, keeping the actual level pairs fixed. The data term $L_{\mathrm{data}}$ is the mean squared error of standardized UCS predictions. Equation (2) combines the data term with the three penalties; no outer-test data enter the training loss.

Five weight triplets $\left(\lambda_{\mathrm{WB}},\lambda_{\mathrm{Cycle}},\lambda_{\mathrm{pos}}\right)$ were considered: (0,0,0), (0.01,0.01,0.001), (0.1,0.1,0.01), (0.1,0.01,0.01), and (0.01,0.1,0.01). Inner three-fold grouped MAE selected these weights independently within each outer training partition.

The retained model-selection summary lists $\left(\lambda_{\mathrm{WB}},\lambda_{\mathrm{Cycle}},\lambda_{\mathrm{pos}}\right)=\left(0.1,0.1,0.01\right)$ for outer Fold 1 and (0,0,0) for Folds 2–5. This is an architecture-matched comparison by design, since only the penalty terms distinguish the specified PINN objective from the BPNN objective at otherwise identical settings. However, full candidate-wise inner-fold records and independent training checkpoints are not retained for this revised configuration; the listed selections cannot, therefore, be re-audited from the surviving artifacts. The penalties are squared hinge losses, which contribute zero gradient whenever their margins are satisfied. Zero post-training violations on a finite diagnostic grid cannot establish that the losses and their gradients were zero during training, nor establish that they were nonzero. Without component-wise penalty histories, the training-time influence of regularization in the original model-selection run remains undetermined.

**Figure 10:**
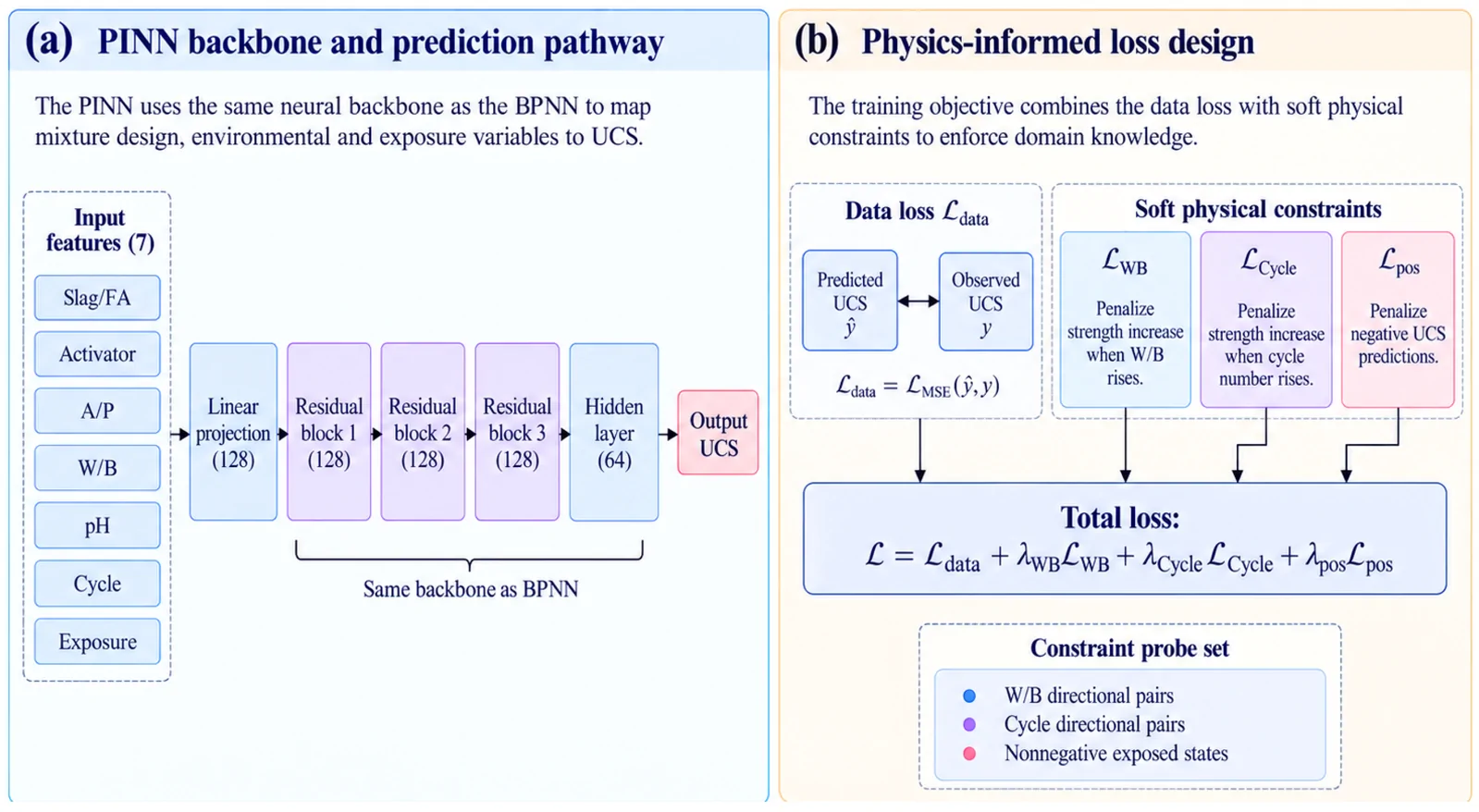
PINN architecture and directional physical soft constraints.

As a separate prospective mechanism check, we ran paired, fixed-weight training trajectories with the reconstructed seven-input, 128-unit residual backbone. For each of the five outer training partitions and seeds 42 and 52, the zero-penalty and nonzero-penalty objectives used identical initial weights, batches, normalization, AdamW learning rate (0.001), weight decay ($10^{-5}$), and 250 full-batch updates. The nonzero triplet was (0.1,0.1,0.01); all counterfactual anchors came from each outer training partition. Three unweighted hinge components and the combined weighted penalty were recorded at every update. No early stopping or tuning was imposed in this mechanism-only diagnostic; its trajectories neither recreate the archived nested-CV candidate searches nor replace the primary OOF predictions. Both prospective diagnostics used a separately reconstructed implementation (PyTorch 2.10.0+cpu), whereas the software listed in Section 3.4 describes the historical primary assessment. Figure 10 summarizes the intended objective and shared backbone.

(1) $$L_{\mathrm{data}}=\frac{1}{n}\sum_{i=1}^{n}{\left(y_{i}-{\hat{y}}_{i}\right)}^{2}$$

(2) $$L_{\mathrm{PINN}}=L_{\mathrm{data}}+\lambda_{\mathrm{WB}}L_{\mathrm{WB}}+\lambda_{\mathrm{Cycle}}L_{\mathrm{Cycle}}+\lambda_{\mathrm{pos}}L_{\mathrm{pos}}$$

### 3.6. Ordered Deterioration Sequences and Shared Baselines

UCS testing is destructive: successive cycle levels represent different specimens, not repeated tests on one evolving specimen. The five positions (Cycle = 0, 1, 5, 10, 15) encode ordered experimental conditions, not measured longitudinal damage. The 159 paths comprise 53 mixtures under three nominal pH exposures; their 795 observation mappings represent 689 distinct UCS records, with the common baseline given one-third weight on each pH path. Missing positions remain masked and duplicate-stage observations remain distinct labels. At an exposed step, the formulation and pH are held fixed along a path, and prior Cycle levels follow the study design; therefore, previous covariates can be reconstructed from the current condition without any measured earlier UCS values.

**Figure 11:**
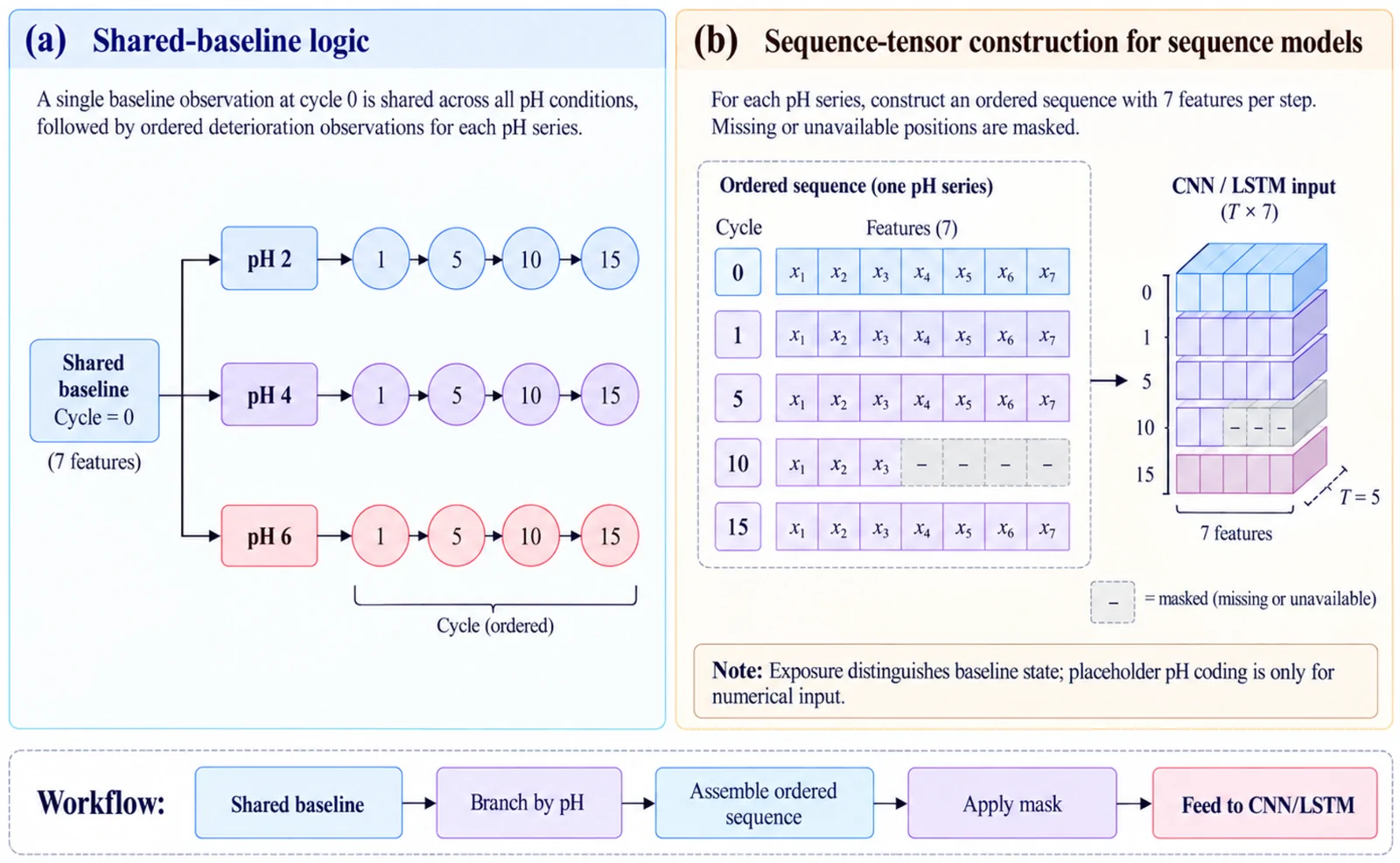
Construction of ordered deterioration sequences and treatment of the shared baseline.

To answer the reviewer using verifiable predictions, we performed a new, structure-matched contextual ablation distinct from the unrecoverable older one-step report. Both variants retain all five input positions and the same two-layer causal CNN (32 channels, kernel size 3) or one-layer LSTM (32 hidden units), with supervision at the mapped observation step. In the masked variant, positions strictly preceding the evaluated step are replaced by zero after fitting-only standardization (the corresponding training-fold means), while the current position, target, sample weights, causal structure, and sequence length remain unchanged. As the masking is target-specific, each label is evaluated using its own five-position input; future positions are inaccessible to either causal model. Five common held-out Mix_ID outer folds and three inner mixture-grouped folds were retained. For each variant independently, learning rates 0.001 and 0.003 were compared by mean separated early stopping (patience 18), followed by a two-seed (42, 52) outer-training refit at the median selected epoch count. All 689 unique outer-test predictions were saved and evaluated once, with the shared baseline predictions averaged across its three mapped paths. Paired uncertainty used 10,000 common resamples of 53 complete mixtures. This supplementary run, with a separately declared training budget, is not an exact rerun of the historical Table 7 CNN/LSTM training or a claim about a single specimen’s time history. Figure 11 summarizes sequence construction.

### 3.7. Evaluation Metrics and Performance Statistics

The coefficient of determination (R^2^), root-mean-square error (RMSE), and mean absolute error (MAE) were computed directly from each model’s 689 held-out mixture-grouped out-of-fold predictions. The pooled estimates are reported in the principal comparison table, together with the fixed outer-fold protocol. RMSE and MAE are expressed in MPa. R^2^ quantifies variation explained under the specified held-out mixture task. Equations (3)–(5) give the definitions.

Historical random-CV values are excluded from the main comparison because their fixed-hyperparameter selection provenance is unavailable; they must not be conflated with nested GroupKFold results.

(3) $$R^{2}=1-\frac{\sum_{i=1}^{n}{\left(y_{i}-{\hat{y}}_{i}\right)}^{2}}{\sum_{i=1}^{n}{\left(y_{i}-\bar{y}\right)}^{2}}$$

(4) $$\mathrm{RMSE}=\sqrt{\frac{1}{n}\sum_{i=1}^{n}{\left(y_{i}-{\hat{y}}_{i}\right)}^{2}}$$

(5) $$\mathrm{MAE}=\frac{1}{n}\sum_{i=1}^{n}\left|y_{i}-{\hat{y}}_{i}\right|$$

Nested grouped OOF predictions were used to quantify evaluation uncertainty while respecting mixture-level dependence. A paired mixture-cluster bootstrap resampled the 53 Mix_ID groups with replacement 10,000 times (random seed 20261013), retaining all 13 observations per selected mixture. Pooled R^2^, RMSE, and MAE were recomputed on each replicate; 2.5th and 97.5th percentiles give descriptive 95% intervals. Model comparisons used the same resampled mixture IDs for paired differences. These conditional intervals describe uncertainty in the existing OOF predictions, not the full variability of retraining or extrapolation beyond the studied design.

The results section presents held-out prediction errors by outer GroupKFold fold rather than training or validation losses recorded during optimization. The fold-wise MAE and RMSE are presented for DT, RF, SVR, XGBoost, PINN, BPNN, CNN, and LSTM. Each fold includes only completely held-out mixtures. Table 7 is calculated from all 689 pooled OOF observations; hence its pooled RMSE need not equal the unweighted mean of five-fold RMSE values. These fold-error plots summarize out-of-fold performance, not model-convergence histories or the separate sequence-ablation experiment.

### 3.8. SHAP and Partial Dependence Analysis

SHAP and PDPs were used to interpret model predictions. Based on Shapley values, SHAP quantifies feature contributions to individual predictions and overall model output within a unified additive framework [22]. PDPs describe the average relationship between a selected feature and the model prediction by marginalizing over other variables, an approach developed within the gradient-boosting literature [30]. Both methods describe associations learned by a model rather than causal relationships. Accumulated local effects (ALE) offer an alternative when correlated inputs make marginal PDP extrapolation problematic [39]; ALE was not implemented in this study. Previous construction-material studies have used XGBoost and SHAP to explain strength predictions and interactions and to support mixture screening [16,17,18]. Deep learning combined with SHAP and two-dimensional PDPs has also been applied directly to one-part geopolymer-stabilized soil [19]. The present study extends this approach through cross-model comparison to assess whether the inferred relationships are stable.

SHAP decomposes a model output into feature contributions using Shapley values from cooperative game theory, providing local additivity and interpretable global summaries. Tree models were analyzed using exact TreeSHAP. SVR, BPNN, and PINN were analyzed using the SHAP permutation explainer, maintaining a common model-agnostic implementation. SHAP was used to examine global importance, defined by mean absolute contributions, and effect direction. Exact feature-interaction decomposition was restricted to tree models; no equivalent decomposition was claimed for the deep-learning models. Exposure is a technical encoding variable rather than a physical factor, so its contribution was reported separately and excluded from the ranking of the six physical factors. For 1D-CNN and LSTM, whose inputs are sequence tensors, predictions were first mapped to a pointwise prediction function by selecting the output at the observation’s corresponding sequence step. Feature contributions were then calculated with the permutation explainer and aggregated across sequence steps into seven-dimensional importance summaries. All eight models were, therefore, evaluated within the SHAP framework.

For feature j, global SHAP importance was defined as the mean absolute contribution, $I_{j}=\frac{1}{n}\sum_{i=1}^{n}\left|\phi_{ij}\right|$. Because different models used different SHAP explainers, each model’s importance values were normalized by its own maximum for cross-model comparison. The analysis emphasizes ranking and direction rather than directly comparing raw absolute SHAP magnitudes.

Partial dependence plots (PDPs) describe predicted responses, not causal effects. Section 4.7 compares the median and 25th–75th percentile band of six pointwise models with XGBoost. The single-factor pH and Cycle panels display only the exposed design levels pH = 2, 4, 6 and Cycle = 1, 5, 10, 15; baseline placeholders are excluded from those displayed levels. The underlying previously archived PDP predictions were not refitted for this display adjustment. For two-factor analysis, a separate explanatory XGBoost regressor was fitted with n_estimators = 300, learning_rate = 0.10, max_depth = 3, subsample = 0.8, random_state = 42, and tree_method = hist (n_jobs = −1; verbosity = 0). These fixed settings differed from the optimal outer-fold parameters. The archived SHAP/PDP implementation excluded eight sequence-incomplete observations, leaving 681 records for this explanation fit; the nested GroupKFold evaluation used all 689. This fit was used only for interpretation, never to obtain Table 7 metrics. The factor pairs were Slag/FA × Cycle, W/B × pH, and Slag/FA × W/B. Each pair was evaluated at actual experimental design levels, while predictions were averaged across the empirical distribution of the remaining input factors.

To avoid confounding physical interpretation with zero-cycle baselines and their numerical pH placeholder, all three two-factor PDPs were calculated within the acid-exposed domain, Exposure = 1. The evaluated levels were Slag/FA = 2.34, 4, and 9; W/B = 0.5, 0.7, and 0.9; pH = 2, 4, and 6 and Cycle = 1, 5, 10, and 15. Surface height and color jointly represent the partial dependence of predicted UCS, and contours on the base plane aid visualization of gradient direction. The surfaces visually connect actual design nodes; smooth transitions between discrete levels are not interpreted as continuous physical thresholds. Both one-factor and two-factor PDPs describe learned associations rather than causal effects.

## 4. Results and Discussion

### 4.1. Dataset Characteristics

**Figure 12:**
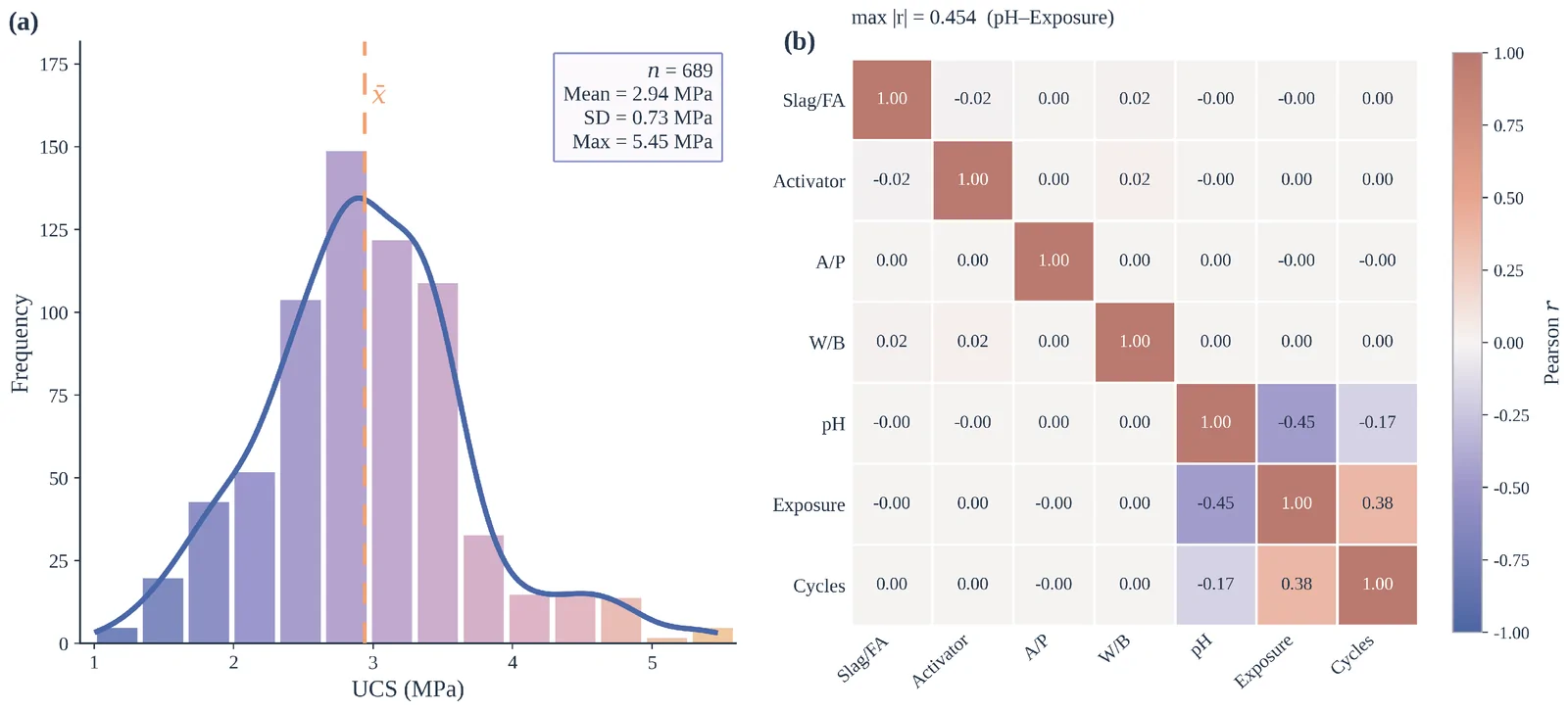
UCS distribution and input-variable correlation matrix for the 689 observations (**a**,**b**).

Between 2.5 and 3.5 MPa. Seven records exceeded 5 MPa; these values were retained and flagged, although independent replicate checks remain unavailable. Figure 12 presents the UCS distribution and input-correlation matrix. Pearson coefficients alone do not capture that Exposure is completely determined by Cycle (Exposure = 1 when Cycle > 0). It adds no independent physical information, and feature attributions must be interpreted with this redundancy in mind.

### 4.2. Validation Protocol and Historical Parameter-Selection Risk

An earlier analysis compared random five-fold cross-validation with mixture-grouped splitting using one fixed hyperparameter configuration for each of four conventional learners. On review, all four fixed-configuration grouped scores exceeded the corresponding estimates from the subsequently implemented nested mixture-grouped evaluation. This pattern warrants scrutiny because the surviving files do not establish when those fixed hyperparameters were chosen or whether the full dataset, its cross-validation scores, or held-out mixture outcomes influenced their selection. Consequently, the historical fixed-configuration estimates cannot be treated as unbiased held-out performance or used to assess the relative merits of fixed versus nested tuning. Their originally displayed comparison table and plot have been removed, rather than explained away or presented as evidence that nested tuning necessarily underperforms.

This provenance gap is a risk of selection bias, not proof that information leakage occurred. A difference between historical and current scores can also reflect model implementation, software versions, data preprocessing, optimization, and other protocol changes. Without a complete historical selection record, the contributions of these factors cannot be identified or isolated, and a controlled random-versus-grouped score contrast is not claimed. The earlier random-fold figures are, therefore, not included in the quantitative comparisons of this revision.

The primary analysis instead uses five outer GroupKFold partitions by complete Mix_ID and independent three-fold mixture-grouped tuning within each outer training partition. The four conventional learners have preserved outer-fold prediction files, parameter selection summaries, and fold-specific search results, allowing the training-versus-test separation and principal OOF metrics to be checked. The common held-out mixture evaluation is reported for all eight learners in Table 7; separate paired uncertainty comparisons are presented subsequently using the same fixed OOF predictions. This is the evidence base used for the predictive conclusions below.

### 4.3. Predictive Performance of the Eight Models

**Figure 13:**
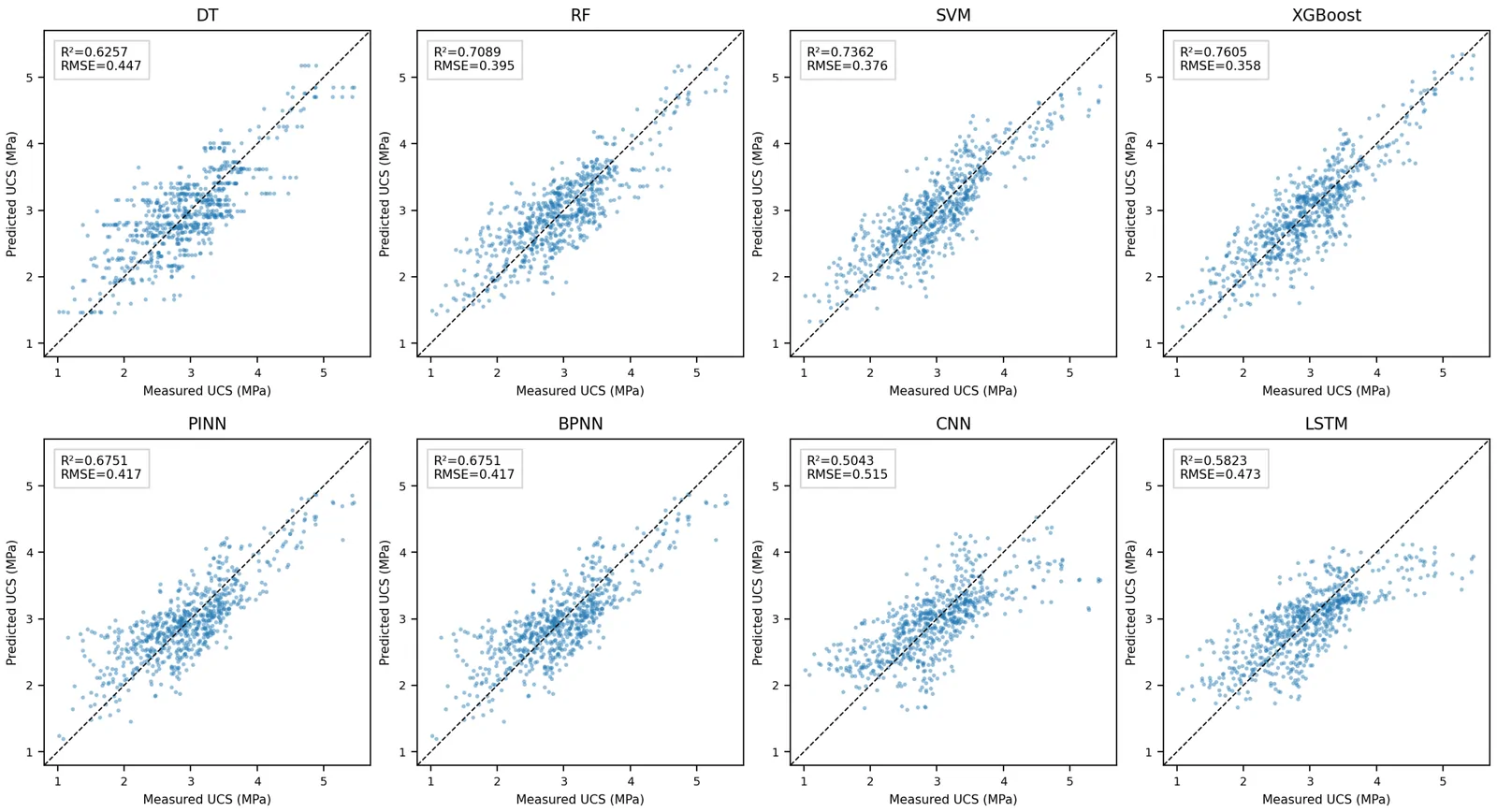
Measured versus predicted UCS: nested mixture-grouped OOF estimates (689 records).

**Figure 14:**
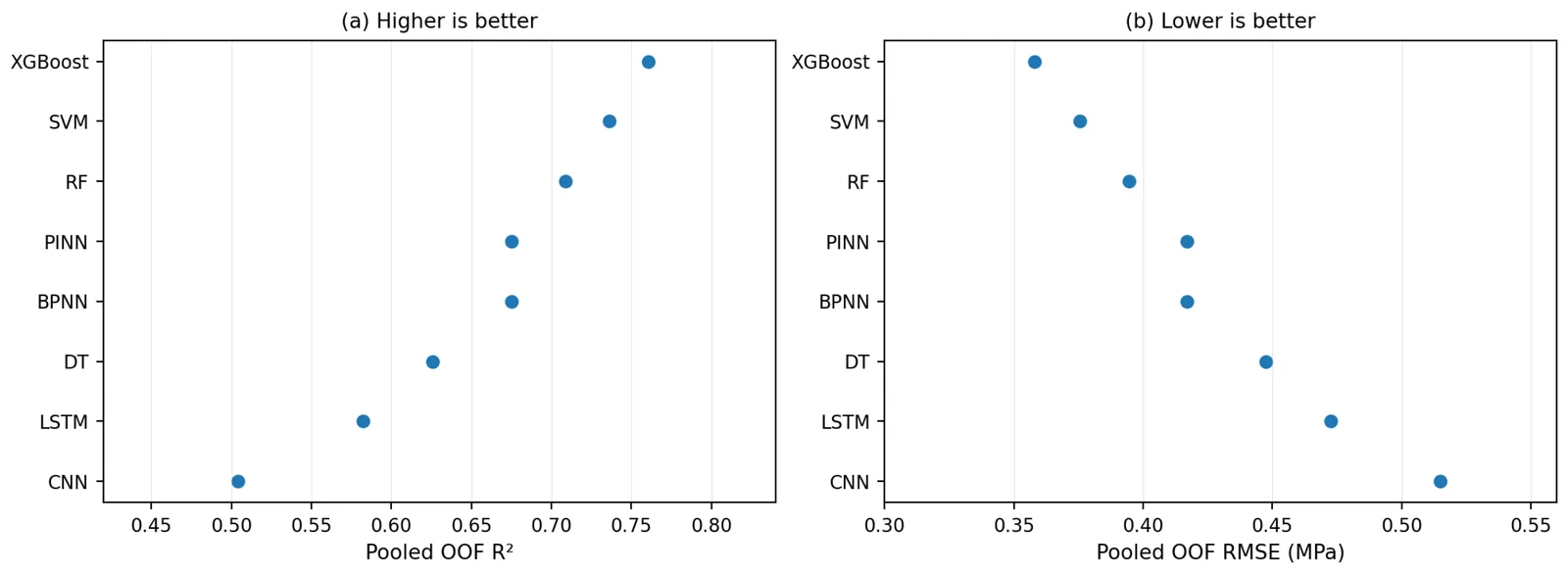
Eight-model rankings under nested GroupKFold based on pooled R^2^ and RMSE.

The eight models were evaluated with identical outer five-fold mixture-grouped partitions and model-specific three-fold grouped hyperparameter selection. All 689 original observations received one outer-test prediction. Table 7 presents the pooled OOF $R^{2}$, RMSE, and MAE for all eight models. XGBoost had the highest $R^{2}$ (0.7605) and lowest RMSE (0.3579 MPa), followed by SVM ($R^{2}=0.7362$; RMSE = 0.3756 MPa). The $R^{2}$ values for RF, PINN, BPNN, DT, LSTM, and CNN were 0.7089, 0.6751, 0.6751, 0.6257, 0.5823, and 0.5043, respectively. These are differences in point estimates, not evidence of statistically established superiority. Held-out-mixture performance varied by fold, consistent with the limited number of independent mixture groups and discrete experimental factor levels. Figure 13 and Figure 14 have been updated to display the eight nested-CV OOF predictions and corresponding rankings.

Exposure redundancy check. A matched XGBoost re-fit compared six inputs (excluding Exposure) with all seven, using identical outer/inner mixture-grouped folds, 16-setting tuning grid, and seed 42. Both versions selected the same per-fold settings and gave identical results (XGBoost 3.1.3). All five saved primary XGBoost models also made zero splits on Exposure. The difference between software runs is not a feature-ablation effect. This check establishes redundancy for XGBoost but not the other seven-input models.

PINN and BPNN were specified with the same residual architecture, inputs, optimizer, and seeds, with nonzero soft-penalty weights permitted only for PINN. The reported post-training finite-grid audit comprised 1268 W/B counterfactual pairs, 477 adjacent-cycle pairs (1 to 5, 5 to 10, and 10 to 15), and 636 exposed-condition strength probes per model, with 0.02 MPa tolerance. It found no violations for either model on those checks. The retained 689-record OOF prediction files are numerically identical, rather than merely having equal rounded metrics. These observations demonstrate no measurable incremental advantage of the penalized configuration: there was no observed BPNN violation on the audited grid for a penalty to correct. Identical saved predictions alone do not establish separate optimization histories, and final grid compliance cannot determine whether penalty gradients acted at earlier training stages. Neither global monotonicity nor the efficacy of penalties in a genuinely violation-prone setting has been demonstrated.

CNN and LSTM used ordered five-position condition paths rather than specimen-level longitudinal time series. The 159-path representation, valid-position masks, and observation mapping preserved all 689 held-out test labels, including the shared baseline. The pooled $R^{2}$ values for CNN and LSTM were 0.5043 and 0.5823, respectively. Neither exceeded the leading pointwise learners on this unseen mixture task. Because earlier covariates are prescribed design levels rather than newly measured historical states, performance here cannot substantiate patient- or specimen-specific trajectory learning, although different sequence data could warrant a separate assessment.

Earlier random-fold results reflected an interpolation-oriented task, but their original parameter-selection provenance cannot be verified and their values are omitted from the present model ranking. The preserved mixture-grouped OOF results, obtained with outertest separation and training-only inner tuning, support preliminary screening but not engineering use without independent validation.

Four neural models returned 0.5043–0.6751 under the same group-separated outer folds. Differences between the two categories should not be interpreted as proving that either family always generalizes better; the comparison concerns 53 independent mixtures from one experimental design.

The archived inner-CV summary for the five tested PINN weight triplets reports mean MAEs of 0.346617–0.346859 MPa, with a maximum reduction of only 0.000242 MPa (about 0.07%) against zero weights. The outer fold 1 nonzero choice and folds 2–5 zero choices remain historical summary statements: matched candidate-level logs were not preserved, so their precise score variation cannot be attributed retrospectively. If a penalty is zero at every training update and all other training conditions are truly matched and deterministic, changing its coefficient cannot change a fitted prediction. The original final zero-violation audit does not test this necessary premise.

In the new fixed-trajectory mechanism check, the combined nonzero-weight penalty (over the 10 trials) was activated for 99–157 of the 250 logged updates. Matched zero- and nonzero-weight parameter vectors differed by at most 0.0396 in absolute coefficient value at epoch 250 (across trials), and the largest observed weighted-penalty value was 0.002651 in standardized loss units. This directly establishes that the newly implemented objective can activate during training and diverge from its zero-penalty control. It does not demonstrate that the missing original candidate trajectories did so, isolate the cause of their minute inner-CV MAE spread, or demonstrate predictive superiority. The archived PINN and BPNN have identical retained OOF predictions ($R^{2}=0.6751$; RMSE = 0.4168 MPa for both), and both have zero reported final-grid violations. RQ2, therefore, has no demonstrated incremental performance or final-grid compliance benefit in the primary experiment, even though active soft penalties are possible in the prospective diagnostic.

The prospectively run, fixed-length ablation yielded an independently verifiable comparison in which identical test mixtures, mapped UCS labels and causal network topology were used for each pair; only access to preceding design covariates differed. For LSTM, the corresponding values were 0.5877 (0.4695 MPa) and 0.6123 (0.4553 MPa). The paired mixture-bootstrap contrast, defined as RMSE(full) minus RMSE(masked), was +0.0422 MPa (95% CI 0.0157 to +0.0690) for CNN and +0.0142 MPa (95% CI −0.0069 to +0.0368) for LSTM. Thus, the available five-position context did not improve the present CNN; the LSTM interval included zero. Table 8 distinguishes these new diagnostic estimates from the historically archived performance in Table 7. These are conditional intervals for fixed out-of-fold predictions; they do not include retraining uncertainty or fully span architectural choices. The search varied learning rate and early-stopping duration but not model width or depth. Because preceding covariates are design-determined and not separately measured specimen states, the experiment cannot establish a causal history effect beyond the available conditions. The pointwise BPNN benchmark remains an independent comparator, not a capacity-matched context control. The main-model outer-fold error plots are distinct from this supplementary sequence ablation. The older one-step summary, which changed effective network computation and lacks saved OOF output, has been withdrawn as quantitative evidence.

The eight-model comparison, therefore, reflects the shared nested mixture-grouped test protocol and verified OOF predictions. The results support cautious assessment of new-mixture prediction rather than extrapolative guarantees.

**Figure 15:**
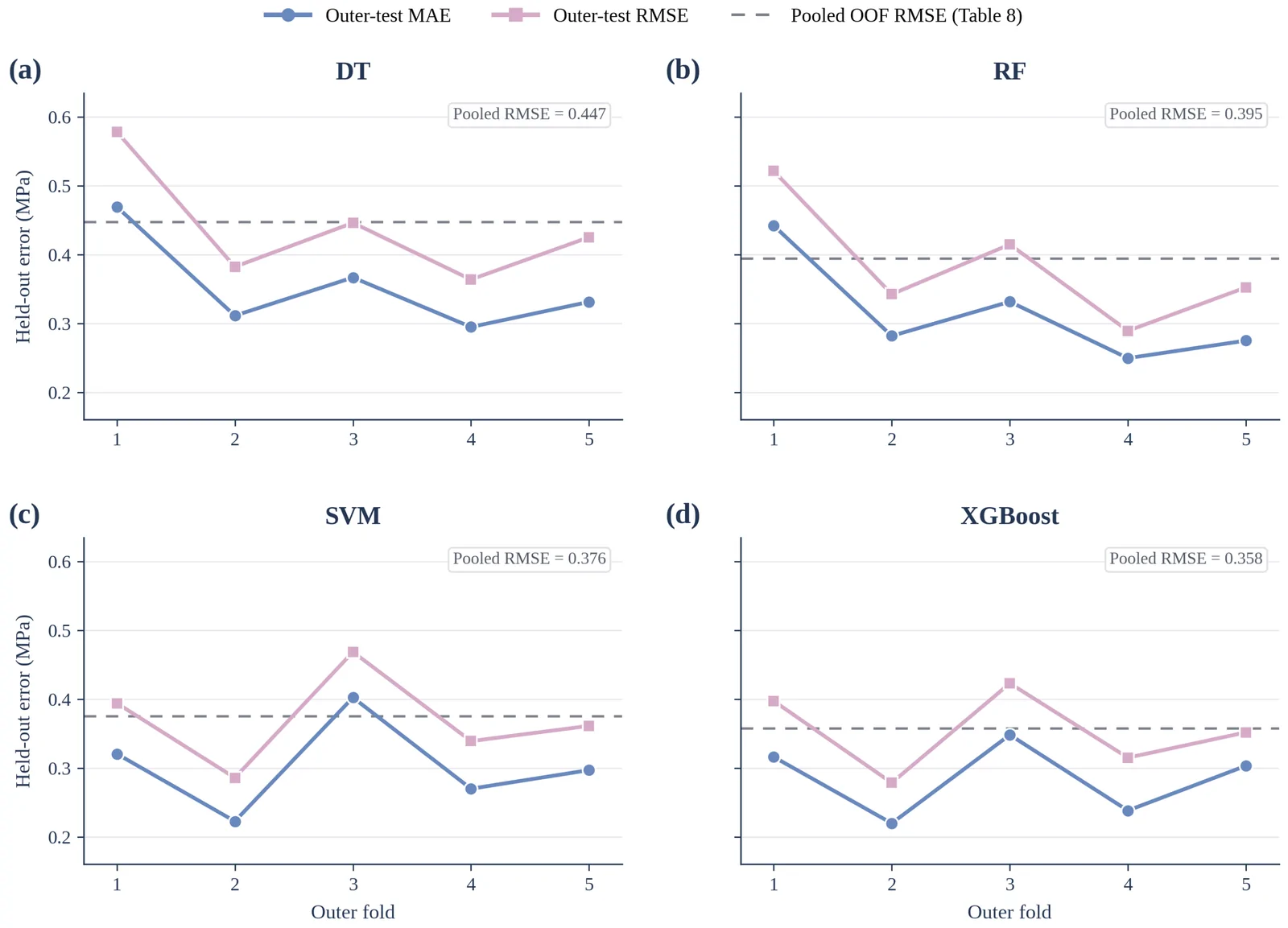
Outer-test MAE and RMSE for (**a**) DT, (**b**) RF, (**c**) SVM, and (**d**) XGBoost. Dashed lines indicate the pooled OOF RMSE from all 689 held-out records (Table 7).

The outer-fold errors of the conventional models are shown in Figure 15.

**Figure 16:**
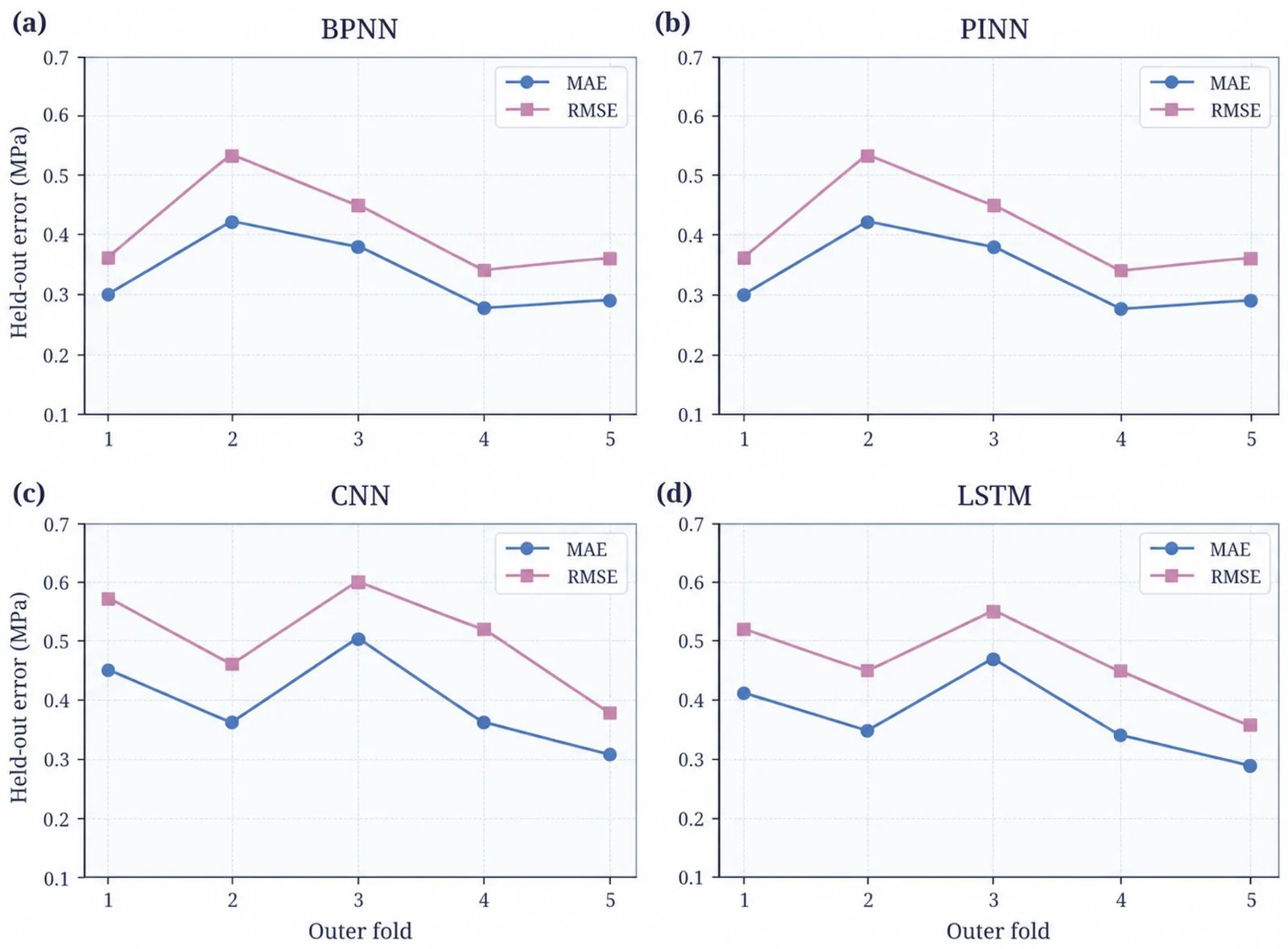
Fold-wise held-out MAE and RMSE for four neural models in nested mixture-grouped CV: (**a**) BPNN, (**b**) PINN, (**c**) CNN, and (**d**) LSTM.

The outer-fold errors of the neural models are shown in Figure 16.

### 4.4. Descriptive Statistics of Performance Differences

Table 9 reports paired comparisons of XGBoost with each of the other seven learners on the identical 689 held-out observations. We applied 10,000 nonparametric bootstrap resamples of the 53 complete mixtures (seed 42), preserving every record within each selected Mix_ID and using identical resamples for both members of each comparison. Differences were defined as $\Delta R^{2}=R_{\mathrm{XGBoost}}^{2}-R_{\mathrm{comparator}}^{2}$ and $\Delta \mathrm{RMSE}={\mathrm{RMSE}}_{\mathrm{XGBoost}}-{\mathrm{RMSE}}_{\mathrm{comparator}}$. The 2.5th and 97.5th percentiles of each paired-bootstrap difference provided its descriptive 95% interval. The confidence intervals of RF and SVM overlap zero for both metrics; the other comparisons exclude zero narrowly or more clearly under this fixed-prediction, mixture-cluster bootstrap. Such intervals do not establish broad model-family superiority, account for hyperparameter/model-selection multiplicity, include training-model refitting, or establish external field validity. The PINN and BPNN comparisons have identical intervals because their 689 OOF predictions coincide. Historical fixed-parameter comparisons were withdrawn owing to unresolved parameter-selection provenance.

### 4.5. Sensitivity to Training-Set Size

**Figure 17:**
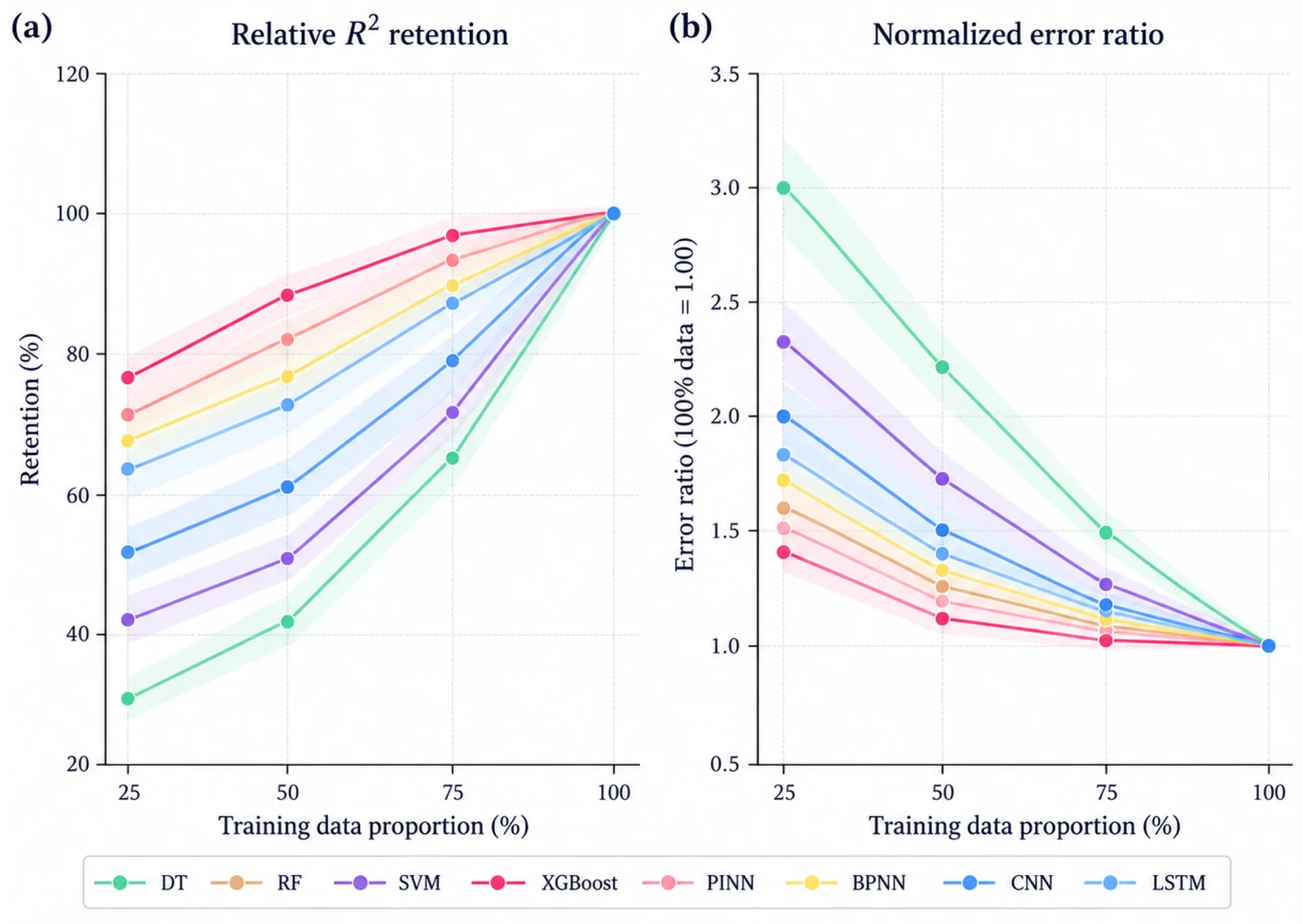
Relative R^2^ retention and normalized error ratio at different training-data fractions.

Training-set-size sensitivity was examined only as a supplementary stability analysis, assessing relative performance degradation as training information decreases. To avoid duplicating or conflicting with the final absolute performance in Table 7, Figure 17 normalizes each model by its own result with 100% of the training data. Relative R^2^ retention is defined as R^2^(f)/R^2^(100%) × 100%, and the normalized error ratio as Error(f)/Error(100%). The endpoints at 100% training data are, therefore, 100% and 1.00, respectively. The figure compares sensitivity to reductions in training data rather than redefining the final R^2^ or RMSE of any model.

### 4.6. Global SHAP Importance and Cross-Model Consistency

**Figure 18:**
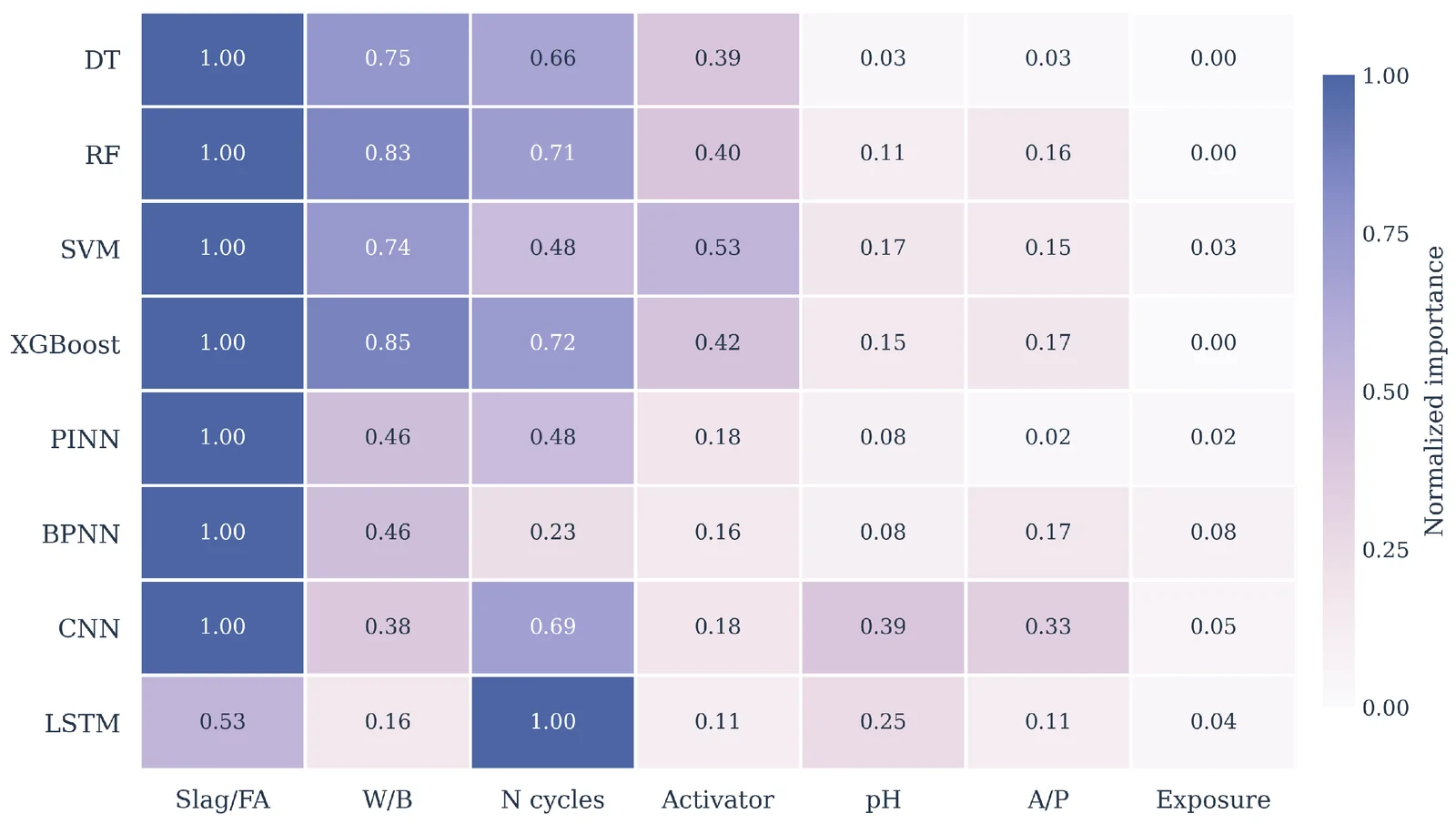
Normalized global SHAP importance across the eight models.

Figure 18 compares normalized global SHAP importance across the eight models. Because the explainers and internal representations differ among tree models, SVR, and deep-learning models, raw SHAP magnitudes are not treated as directly comparable quantitative conclusions. Instead, the analysis emphasizes importance rankings, effect directions, and whether these patterns recur across models. Slag/FA, W/B, and Cycle form the most stable group of high-importance factors. Activator, pH, and A/P contribute less globally but may still have appreciable local effects through conditional interactions. Exposure primarily identifies baseline conditions and has less direct physical interpretability than the six experimental factors.

Most models rank Slag/FA first or second, with W/B and Cycle also consistently among the leading factors. Some sequence models place greater relative weight on Cycle. This recurrence in rankings provides stronger evidence of robustness than any individual normalized importance value. Cross-model consistency is, therefore, interpreted as the repeated identification of important factors and effect directions, rather than agreement on identical SHAP values.

XGBoost OOF SHAP ranked Slag/FA, W/B, and Cycle as the three leading factors. To probe the design imbalance, SHAP values from the unchanged five held-out XGBoost fits were re-aggregated after excluding the unmatched NaOH mixture (13 records), yielding 52 complete matched mixtures. The full seven-feature importance ordering was unchanged. This is a sensitivity test of the explanation reference population, not model retraining and not evidence about the unmeasured sodium-silicate formulation. SHAP rankings remain predictive associations rather than causal conclusions.

Cross-model consistency is examined at three levels: importance ranking, effect direction, and response shape. The specified PINN objective includes priors for W/B, Cycle, and nonnegative strength, so a directional trend in these factors could in principle reflect a training constraint rather than independent evidence. However, the available training record does not establish active penalty gradients, and the archived PINN/BPNN OOF predictions coincide. Directional agreement must, therefore, not be attributed to effective regularization; independent evidence is better sought in agreement among purely data-driven learners and variables not targeted by the priors (Slag/FA, Activator, A/P, and pH).

For PINN, the presence of a soft-constraint term in a loss function must be distinguished from evidence that the term materially influenced learned responses. Neither SHAP directions nor PDP shapes can identify penalty activation in the absence of matched training trajectories or an observed PINN–BPNN difference. We, therefore, describe W/B and Cycle trends as model associations only; we do not label them constraint-induced. Agreement in unconstrained variables (Slag/FA, Activator, A/P, and pH) and among models without directional penalties remains useful descriptive corroboration, not proof of physical regularization effectiveness.

**Figure 19:**
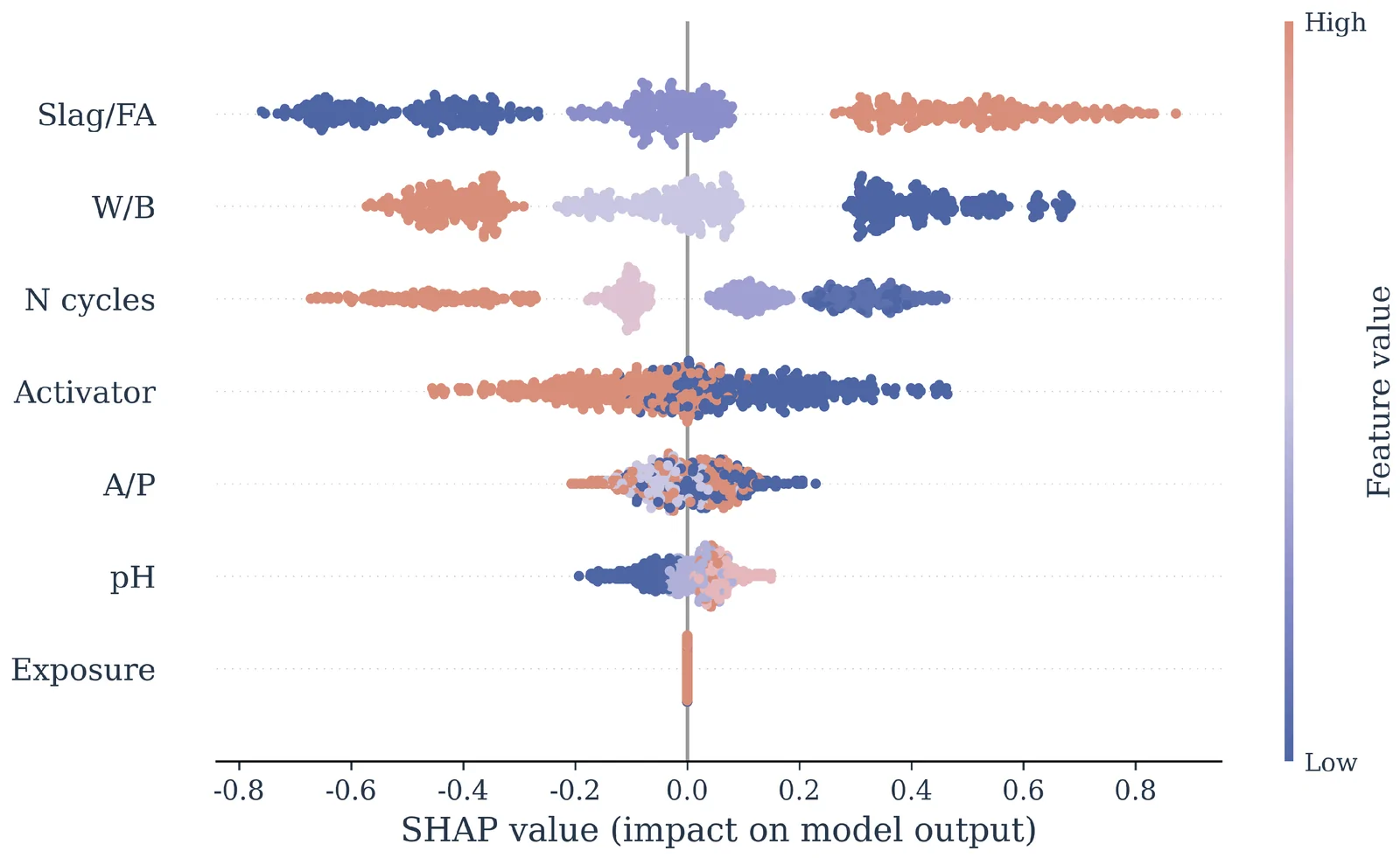
Enhanced SHAP beeswarm plot for XGBoost.

Figure 19 presents an enhanced XGBoost SHAP bee swarm plot of local feature contributions. The horizontal axis gives the SHAP value, indicating a positive or negative contribution to predicted UCS. Each point represents an observation, color encodes the actual feature value from low to high, and slight vertical spreading reveals observation density. The plot simultaneously shows contribution direction, dispersion, and the distribution of high and low feature values across positive and negative contribution regions, complementing the global rankings in Figure 18.

The central answer to RQ3 is thus based on whether high-importance factors, effect directions, and principal response ranges recur across models, rather than whether all eight models assign identical numerical importance. The results support Slag/FA, W/B, and Cycle as the most stable dominant factor group.

### 4.7. Partial Dependence Responses and Transition Ranges

**Figure 20:**
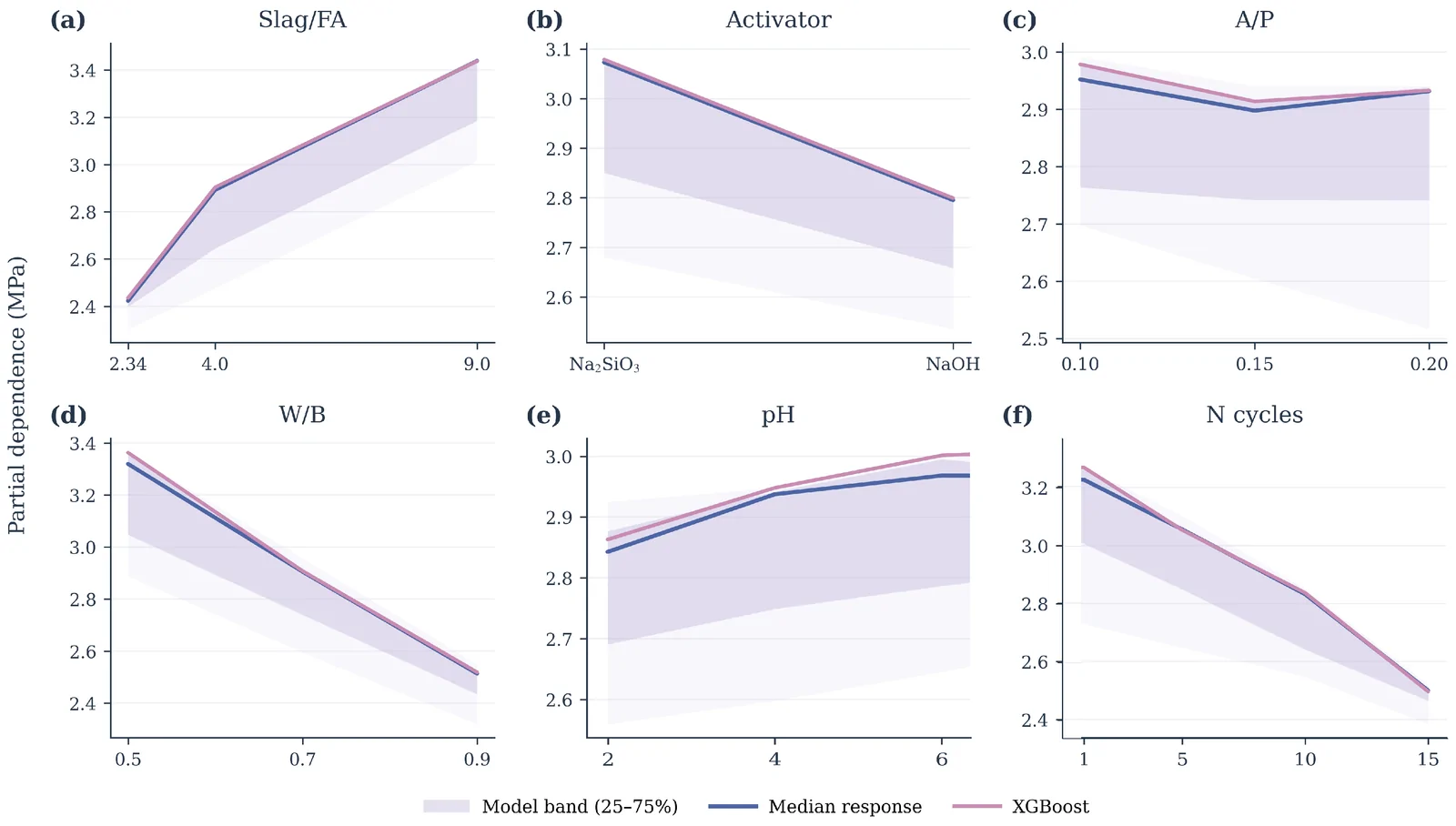
One-factor PDPs across six pointwise models: (**a**) Slag/FA; (**b**) activator class; (**c**) A/P; (**d**) W/B; (**e**) pH; and (**f**) wetting–drying cycles. The blue line is the across-model median, the purple band is the 25th–75th percentile range, and the pink line represents XGBoost. Panels (**e**,**f**) show only the acid-exposed design levels; no exposed-only PDP recalculation was performed.

Figure 20 shows the median PDP response across the six pointwise models (DT, RF, SVM, XGBoost, BPNN, and PINN), the 25th–75th percentile band of model responses, and the XGBoost curve. Predicted UCS generally increases with Slag/FA and decreases across nominal W/B levels within this database. Increasing Cycle produces cumulative deterioration: changes are relatively limited at early stages, followed by more pronounced strength loss during intermediate and later cycles. In Figure 20, the pH and Cycle panels omit the baseline-only levels pH = 7 and Cycle = 0. Their remaining ordinates are unchanged; this is a display-domain restriction, not a new exposed-only marginalization. Predicted UCS rises modestly across pH 2, 4, and 6, consistent with prior GGBFS/FA-stabilized soil studies [2,3,9,10].

**Figure 21:**
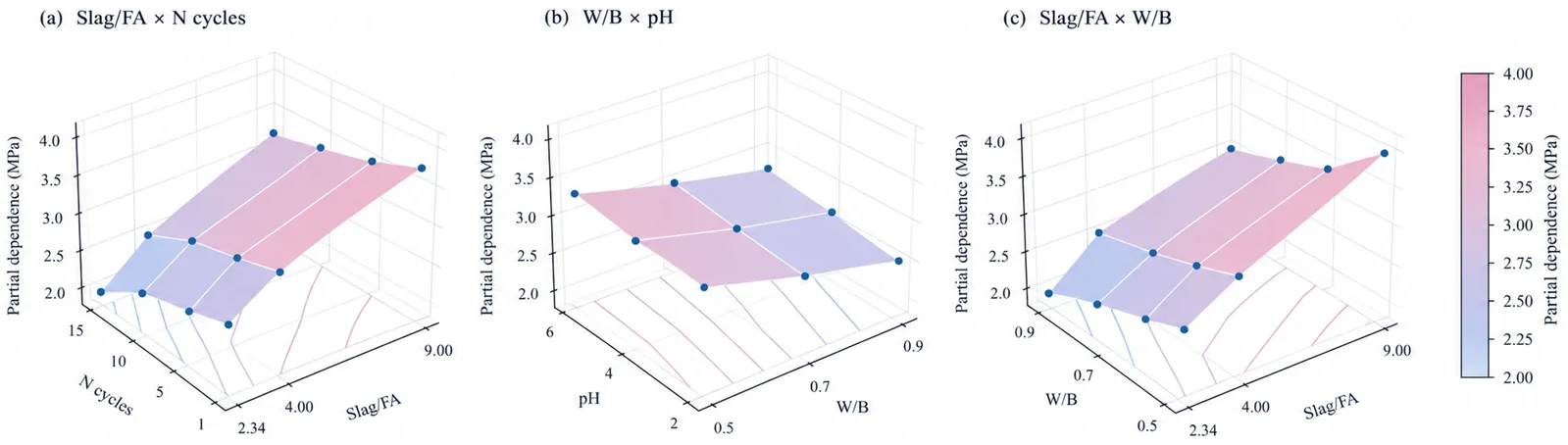
Three-dimensional XGBoost two-factor PDP response surfaces at actual design levels within the acid-exposed domain: (**a**) Slag/FA × wetting–drying cycle number; (**b**) W/B × pH and (**c**) Slag/FA × W/B.

Three materially relevant factor pairs were selected for supplementary analysis, keeping the scope focused rather than extending it to an exhaustive interaction study (Figure 21). In the Slag/FA × Cycle surface, higher Slag/FA corresponds to higher predicted UCS at every cycling stage. As Cycle increases from 1 to 15, the partial dependence values at the three Slag/FA levels decrease from 2.802, 3.215, and 3.745 MPa to 1.860, 2.444, and 3.126 MPa, respectively. Thus, the magnitude of cumulative cycle-induced loss depends on precursor composition. The nominal W/B × pH surface is comparatively smooth, with approximately parallel responses: moving from recorded W/B level 0.5 to 0.9 lowers predicted UCS at pH 2, 4, and 6, whereas increasing pH shifts the surface upward only slightly. This pair is, therefore, dominated by approximately additive main effects. In the Slag/FA × W/B surface, increasing Slag/FA raises predicted UCS at all three W/B levels, but slopes and separations vary with W/B, indicating conditional dependence between precursor composition and water content. Two-factor PDPs consequently supplement the one-factor interpretation by identifying differences in conditional responses; they do not establish universal synergistic mechanisms or precise critical thresholds.

### 4.8. Conditional Response Domains of the Activator Systems

To examine how the relative performance of the two activators depends on exposure conditions, XGBoost was used to calculate ΔUCS = UCS(sodium silicate)-UCS(NaOH) over the (pH, cycle number) domain separately at Slag/FA = 2.34, 4, and 9. Positive ΔUCS indicates higher predicted UCS with sodium silicate, while negative ΔUCS favors NaOH. The target Slag/FA, pH, Cycle, and activator type were set to specified values, and predictions were averaged over the empirical distribution of the remaining variables to obtain conditional differences.

**Figure 22:**
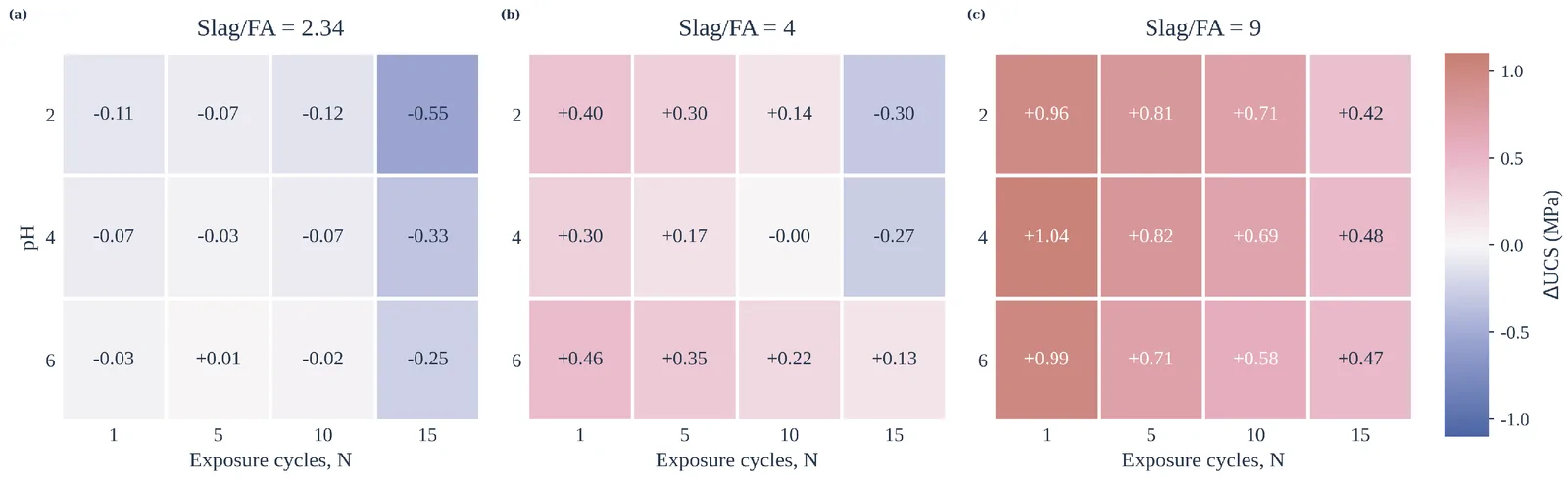
Conditional response domains of the activator systems at (**a**) Slag/FA = 2.34, (**b**) Slag/FA = 4, and (**c**) Slag/FA = 9. $\Delta \mathrm{UCS}={\mathrm{UCS}}_{\mathrm{silicate}}-{\mathrm{UCS}}_{\mathrm{NaOH}}$ (MPa); negative contrasts favor NaOH.

Figure 22 contrasts activator classes at equal nominal A/P rather than equal Na_2_O-equivalent dosage. NaOH is often favored at Slag/FA = 2.34 and sodium silicate at 9. Near-zero values at the intermediate ratio do not establish crossover thresholds. Across five saved outer-fold XGBoost fits, one of the 12 cells at Slag/FA = 4 changed contrast sign (pH = 6, Cycle = 15; range −0.071 to +0.102 MPa). This between-fit range is not a confidence interval. Separately, observed activator contrasts were paired by composition and exposure. After excluding two mixture groups with incomplete cycle labels, seven valid pairs remained at Slag/FA = 4. At pH = 4 and Cycle = 10, their mean contrast was −0.045 MPa (95% pair-bootstrap interval −0.231 to +0.182 MPa); three of 12 conditional intervals included zero. These observed-data intervals do not quantify Figure 22 prediction uncertainty. One of the 54 planned formulations, sodium silicate at Slag/FA = 2.34, A/P = 0.15, W/B = 0.9, was not observed. Thus, only 26 of 27 activator-matched material contrasts can be computed, including eight of nine at Slag/FA = 2.34. No UCS value was imputed. To check sensitivity to this imbalance without retraining, the unmatched NaOH reference group was excluded when aggregating the five stored outer-fold XGBoost fits. Across the 36 pH-by-cycle activator-contrast cells, the largest absolute shift was 0.0027 MPa, with no sign reversals; the three two-factor PDP surfaces shifted by at most 0.0192 MPa. The held-out XGBoost SHAP ranking was also unchanged under this reference-distribution restriction (Section 4.6). These diagnostics concern unchanged models and the observed reference population; they cannot establish the missing mixture strength or the effect that including new experimental data would have after refitting. Inference near the missing cell remains limited.

The two activators cannot be ranked independently of material composition and service environment. Within the present design space, lower Slag/FA generally favors NaOH, higher Slag/FA generally favors sodium silicate, and intermediate Slag/FA exhibits condition-dependent crossover behavior. Earlier studies likewise show that activator composition and slag content jointly affect reaction development and resistance to aggressive exposure, precluding a universal ranking detached from mixture and environmental conditions [1,7,13,14]. Figure 22 visualizes effect directions and reversal regions within this study’s design space rather than a universal causal law.

### 4.9. Mechanistic Consistency Between SEM Morphology and Model Interpretation

The SEM images in Figure 4, Figure 5 and Figure 6 are representative fields selected to illustrate visible morphology, not statistical measurements of the specimens as a whole. Figure 4 provides the clearest controlled comparison: The activator (sodium silicate), A/P = 0.10, W/B = 0.7, pH = 2, and Cycle = 10 were fixed while Slag/FA increased from 2.34 to 4 and 9. The measured UCS values increased from 1.445 to 2.288 and 3.307 MPa. The selected views exhibit local differences in matrix continuity, particle contacts, and open voids that are directionally compatible with the higher strength and the model-learned Slag/FA response. They do not establish a lower bulk porosity or greater gel production; a different field of view might show different features. Imaging location and specimen heterogeneity may also influence the visual contrasts.

In Figure 5, the pH = 2 field contains conspicuous open regions, the pH = 4 field contains interparticle cracks, and the pH = 6 field includes locally more continuous material alongside defects. These non-identical fields do not establish a monotonic pH-morphology relationship or isolate chemical dissolution from differences in sampling and specimen preparation. Figure 6 depicts more apparent cracks and fragmentation at later wetting–drying stages, broadly consistent with the declining UCS trend along the Cycle direction. Because the test is destructive, images at different cycle numbers represent different specimens rather than the evolution of a single specimen; visible morphology, therefore, does not demonstrate crack initiation or propagation rates.

Previous work on alkali-activated soils and exposure deterioration discusses binder formation, particle bonding, acid attack, and accumulated cracking [1,2,3,9,10,12]. Such studies, including related SEM/XRD reports, offer plausible background rather than direct proof of the mechanisms in the specimens shown here. No replicated field-of-view analysis, calibrated pore-volume statistics, or matched XRD/EDS data are supplied for these particular images. Thus, neither phase identity (including C-(A)-S-H) nor causation between a local SEM feature and a SHAP/PDP response can be inferred. Quantitative tests using multiple fields per specimen, controlled imaging conditions, image-based statistics or porosimetry, and matched phase/compositional measurements would be needed. We, therefore, treat SEM as qualitative context for UCS/model comparisons, not independent mechanistic validation or proof of a causal pathway from the modeled factors to phase formation and strength.

**Figure 23:**
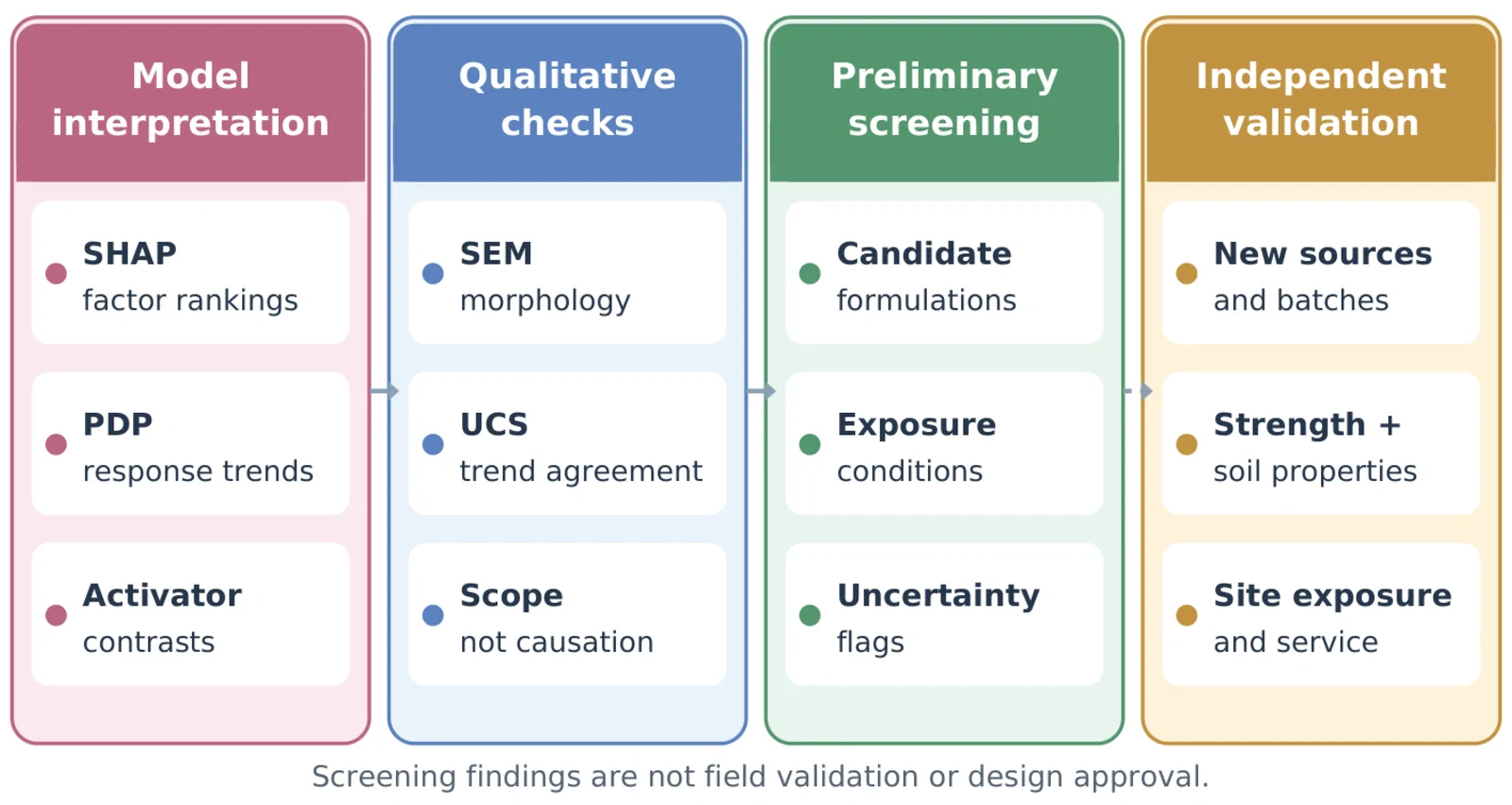
Interpretation and preliminary screening require independent engineering validation before project-specific application.

The interpretation-to-validation workflow is summarized in Figure 23.

## 5. Conclusions

### 5.1. Engineering Findings and Implications

This study assessed UCS after coupled wetting–drying cycles and acid attack using 689 experimental records from 53 slag–fly ash alkali-activated soil mixtures. Its engineering contribution is to connect strength after exposure with mixture composition and environmental conditions, while distinguishing prediction within the sampled design space from prediction for previously unseen mixtures. These associations inform only preliminary formulation screening within the tested material and exposure range, not engineering design.

(1) Prediction accuracy depends on the intended engineering task. In the unified nested mixture-grouped evaluation, XGBoost achieved the highest pooled OOF point estimate ($R^{2}=0.7605$; RMSE = 0.3579 MPa), followed by SVM ($R^{2}=0.7362$; RMSE = 0.3756 MPa). These figures quantify prediction when entire mixture formulations are withheld, rather than interpolation among exposure conditions of known formulations. Previously reported random five-fold scores have been excluded from the current comparison because independence of the historical fixed-parameter selection from evaluation data cannot be established.

(2) Slag/FA, nominal W/B and Cycle were dominant database predictors: higher Slag/FA generally corresponded to higher predicted UCS, whereas higher recorded W/B levels and Cycle corresponded to lower strength. Cycle-related loss also varied with Slag/FA. Since the W/B mass basis is unverified, these within-dataset trends are not transferable quantitative dosage rules.

(3) Activator classes compared at nominal A/P are not equal-alkalinity formulations. Within the tested domain, NaOH generally gave higher predicted UCS at low Slag/FA and sodium silicate at high Slag/FA, with conditional reversals at intermediate ratios. Such trends identify only candidates for experimental comparison, not verified activator preferences or field performance.

(4) The primary PINN and BPNN retain identical 689-record OOF predictions and zero violations in their reported final finite-grid checks; hence no additional predictive or post-training physical-consistency benefit is established. An independently executed matched-initialization diagnostic demonstrates that the reconstructed directional penalties can become nonzero during optimization and alter its parameter trajectory, but cannot recover whether that happened in the original inner-CV candidates or explain their exact tiny MAE spread. A new five-position, target-specific context-masking comparison preserves CNN/LSTM causal architecture, explicitly re-tunes both variants and retains paired OOF predictions. In this supplementary experiment, the full-context CNN performed worse than the preceding-step-masked model; the corresponding LSTM difference was not resolved by its paired mixture-cluster interval. Since the earlier design covariates contain no independently measured prior specimen states, these results do not support a special historical deterioration-learning benefit. Neither diagnostic replaces the primary eight-model scores. Representative SEM figures provide descriptive morphology only.

### 5.2. Applicability and Future Validation

These models are preliminary screening tools limited to one studied soft-soil system and its recorded formulation and exposure ranges, not engineering design or service-life prediction tools. W/B and A/P lack complete batch-level traceability, and the archived historical fixed-parameter score comparison was withdrawn owing to unverifiable parameter-selection provenance. The original PINN candidate histories remain unavailable; the new penalty-activation diagnostic is a separate fixed-trajectory re-implementation and cannot retrospectively determine candidate-specific inner-CV effects. The new structure-matched sequence ablation retains test predictions and grouped tuning outputs, but its restricted hyperparameter grid and different training budget do not make it an exact reproduction of historical CNN/LSTM scores. Any general claims about context benefit require additional datasets with independently measured prior states and external validation. Independent validation is required before engineering application. Transfer to a project requires three stages: (i) test previously unseen mixtures made with independent slag, fly-ash, and softsoil batches from other sites; (ii) verify UCS alongside stiffness, shear strength, permeability, compressibility, and settlement under site-specific loads and groundwater chemistry and (iii) monitor longer-term field exposure to assess whether laboratory acidic wetting–drying cycles reflect service conditions. No calibrated conversion from cycle number to field service years is available. Model uncertainty and predicted activator crossovers should guide targeted confirmation tests, not specify binder dosage, bearing capacity, or service life. SHAP/PDP findings are model associations, while SEM supplies qualitative morphological context; neither constitutes independent engineering validation.

Existing geotechnical studies help define a concrete extension route, but they do not externally validate the present models. Zhang et al. developed a composition-informed machine-learning framework using 990 geopolymer-stabilized-soil mix designs to account for differences among binder systems [40]. At a different engineering scale, Ullah et al. evaluated bottom-ash geopolymer columns for embankment-supported soft-ground improvement, linking material strength to column geometry and bearing-capacity response [41]. Hu et al. also reported experimental confirmation of optimized slag–fly-ash stabilized soil mixtures within their own formulation and curing domain [15]. These examples motivate future work that records source-specific soil index properties and mineralogy, precursor oxide compositions, Na_2_O-equivalent activator dose, silicate modulus, verified binder-to-dry-soil dosage, and the full water mass balance. Such richer measurements would permit external held-out-site testing and prospective mix design, followed by structural- and hydraulic-performance checks on treated ground or model-column systems. In particular, a predicted specimen UCS is not a substitute for settlement, load transfer, bearing-capacity, or in situ durability assessment. The different binder chemistries and loading conditions in the cited studies preclude transferring their results, or the present cross-validation scores, directly to a project without independent tests.

## Figures and Tables

**Table 1:** Main chemical compositions of slag and fly ash.

Material	CaO (%)	SiO_2_ (%)	Al_2_O_3_ (%)
GGBFS	≈53.0	≈37.3	—
FA	≈2.8	≈56.4	≈29.4

**Table 2:** Basic physical properties of the soft soil.

Property	Symbol	Value	Unit
Natural water content	w_n_	≈50.0	%
Liquid limit	wL	43.3	%
Plastic limit	wP	24.6	%

**Table 3:** Experimental mixture design comprising 54 combinations.

Design Factor	Symbol	Levels	No. of Levels
Slag/FA ratio	S/F	2.34, 4, 9	3
Activator type	AT	Sodium silicate powder, NaOH	2
Activator/precursor ratio	A/P	0.10, 0.15, 0.20	3
Water/binder ratio	W/B	0.5, 0.7, 0.9	3
Full-factorial design	—	3 × 2 × 3 × 3	54 mixes

**Table 4:** Combinations of wetting–drying cycles and acidic exposure conditions.

Condition	Exposure	pH	Cycle N	Conditions per Complete Mix
Baseline	0	Not defined (numeric placeholder = 7)	0	1
Acidic wetting–drying	1	2, 4, 6	1	3
Acidic wetting–drying	1	2, 4, 6	5	3
Acidic wetting–drying	1	2, 4, 6	10	3
Acidic wetting–drying	1	2, 4, 6	15	3
Total	—	—	—	13

**Table 5:** Dataset fields for the 689 observations, including Data_Flag audit labels.

Field	Meaning	Type/Encoding	Range/Levels	Use in Modeling
Sample_ID	Unique sample identifier	Integer	1–689	Identifier only
Mix_ID	Complete mix-proportion identifier	Integer	1–53	Grouping variable for GroupKFold
S_F	Slag/FA ratio	Numeric	2.34, 4, 9	Input
Activator	Original dataset class	Categorical text	Na_2_SiO_3_ (code), NaOH	Descriptive field
Activator_code	Encoded activator type	Binary	0 = Na_2_SiO_3_; 1 = NaOH	Input
A_P	Activator/precursor ratio	Numeric	0.10, 0.15, 0.20	Input
W_B	Water/binder ratio	Numeric	0.5, 0.7, 0.9	Input
pH_raw	Raw pH record	Categorical/raw	/, 2, 4, 6	Audit field
pH	Numerical pH used by models	Numeric	2, 4, 6; baseline placeholder = 7	Input
Cycle	Number of wetting–drying cycles	Ordered numeric	0, 1, 5, 10, 15	Input
Exposure	Acid-exposure indicator	Binary	0 = baseline; 1 = exposed	Input
UCS	Unconfined compressive strength	Numeric	1.020–5.453 MPa	Prediction target
Data_Flag	Quality-audit label	Categorical	Normal; Baseline; Sequence_Incomplete; High_UCS_Check	Audit field

**Table 6:** Results of the data-consistency audit.

Audit Item	Observed Result	Count	Treatment in Final Analysis
Designed vs. observed mixes	54 mixes were designed; 53 were observed. Missing mix: S/F = 2.34, Na_2_SiO_3_, A/P = 0.15, W/B = 0.9.	1 mix	No imputation; missing combination reported transparently.
Baseline pH undefined	Cycle = 0 baseline records have pH_raw = “/”.	53 records	Use pH = 7 only as a numerical placeholder and Exposure = 0; placeholder has no physical pH interpretation.
High-UCS records	Seven source UCS values exceed 5 MPa; independent test logs unavailable.	7 records	Retained and flagged as High_UCS_Check.
Incomplete cycle sequences	Two (mix, pH) sequences contain duplicated/missing cycle positions.	8 records	Retained for pointwise models; valid-position masking used in CNN/LSTM sequence construction.
Normal records	No audit issue identified.	623 records	Used directly.
Final modeling database	All 689 source records retained, including flagged cases.	689 records	Used for the nested mixture-grouped evaluation.

**Table 7:** Nested mixture-grouped OOF performance for all eight models (N=689). Bracketed values give 95% mixture-cluster bootstrap intervals (10,000 resamples).

Model	$R^{2}$ [95% CI]	RMSE (MPa) [95% CI]	MAE (MPa)	Rank
DT	0.6257 [0.428, 0.743]	0.4473 [0.395, 0.502]	0.3563	6
RF	0.7089 [0.551, 0.804]	0.3945 [0.343, 0.446]	0.3180	3
SVM	0.7362 [0.601, 0.813]	0.3756 [0.330, 0.422]	0.3032	2
XGBoost	0.7605 [0.610, 0.846]	0.3579 [0.308, 0.408]	0.2856	1
PINN	0.6751 [0.501, 0.772]	0.4168 [0.367, 0.470]	0.3352	4
BPNN	0.6751 [0.501, 0.772]	0.4168 [0.367, 0.470]	0.3352	4
CNN	0.5043 [0.364, 0.592]	0.5148 [0.439, 0.595]	0.3990	8
LSTM	0.5823 [0.466, 0.649]	0.4726 [0.407, 0.540]	0.3705	7

**Table 8:** Structure-matched five-step sequence ablation (nested grouped CV; N=689). $\Delta \mathrm{RMSE}={\mathrm{RMSE}}_{\mathrm{full}}-{\mathrm{RMSE}}_{\mathrm{masked}}$; positive favors masking. RMSE and ΔRMSE are rounded separately; the displayed difference may vary by 0.0001. CIs use 10,000 paired mixture resamples; training differs from Table 7.

Model	Full $R^{2}$	Masked $R^{2}$	Full RMSE	Masked RMSE	ΔRMSE [95% CI]
CNN	0.5104	0.5878	0.5116	0.4695	+0.0422 [0.0157, +0.0690]
LSTM	0.5877	0.6123	0.4695	0.4553	+0.0142 [−0.0069, +0.0368]

**Table 9:** Paired mixture-cluster bootstrap contrasts relative to XGBoost in nested GroupKFold (N=689, 53 mixtures, 10,000 paired resamples). ΔRMSE is XGBoost minus comparator (MPa). CI denotes the 95% percentile interval.

Comparator	$\Delta R^{2}$	95% CI ($\Delta R^{2}$)	ΔRMSE	95% CI (ΔRMSE)	RMSE Reduction
SVM	+0.0243	[−0.0408, +0.0777]	−0.0177	[−0.0577, +0.0250]	4.7%
RF	+0.0515	[−0.0200, +0.1312]	−0.0366	[−0.0859, +0.0128]	9.3%
PINN	+0.0853	[+0.0011, +0.1845]	−0.0589	[−0.1196, −0.0007]	14.1%
BPNN	+0.0853	[+0.0011, +0.1845]	−0.0589	[−0.1196, −0.0007]	14.1%
DT	+0.1347	[+0.0506, +0.2468]	−0.0895	[−0.1467, −0.0320]	20.0%
LSTM	+0.1782	[+0.0816, +0.2525]	−0.1147	[−0.1888, −0.0414]	24.3%
CNN	+0.2562	[+0.1512, +0.3485]	−0.1570	[−0.2425, −0.0747]	30.5%

## Data Availability

The 689 observation-level UCS records analyzed here are supplied in machine-readable form as Supplementary Data S1 accompanying this revised manuscript, with field definitions and audit notes in File S1. The original source observations were documented in Zhang’s 2026 MSc thesis [25]. Upon publication, these supplementary files will be publicly accessible alongside the article. Original specimen-level replicate measurements and laboratory batch sheets are not included. Model scripts and trained-model settings may be requested from the corresponding author, subject to laboratory authorization.

## Supplementary Materials

The following supporting information is supplied with this article at https://www.mdpi.com/article/10.3390/ma19214472/s1: Supplementary Data S1 (CSV), the 689 observation-level UCS records and input variables; Supplementary File S1 (Markdown), field definitions and data-quality audit notes.
